# Supplementary material for: Permeability of the blood-brain barrier through the phases of ischaemic stroke and relation with clinical outcome: protocol for a systematic review
Source: BMJ Open. 2020 Sep 18;10(9):e039280. doi: 10.1136/bmjopen-2020-039280 (PMC7511624; doi:10.1136/bmjopen-2020-039280)
Supplement: Author's manuscript [file bmjopen-2020-039280.draft_revisions.pdf]

BMJ Open is committed to open peer review. As part of this commitment we make the peer review history of every article we publish publicly available.

When an article is published we post the peer reviewers' comments and the authors' responses online. We also post the versions of the paper that were used during peer review. These are the versions that the peer review comments apply to.

The versions of the paper that follow are the versions that were submitted during the peer review process. They are not the versions of record or the final published versions. They should not be cited or distributed as the published version of this manuscript.

BMJ Open is an open access journal and the full, final, typeset and author-corrected version of record of the manuscript is available on our site with no access controls, subscription charges or pay-per-view fees (http://bmjopen.bmj.com).

If you have any questions on BMJ Open's open peer review process please email info.bmjopen@bmj.com

BMJ Open

Permeability of the blood brain barrier through the phases of ischemic stroke and relation with clinical outcome: protocol for a systematic review

Journal:	*BMJ Open*
Manuscript ID	bmjopen-2020-039280
Article Type:	Protocol
Date Submitted by the Author:	13-Apr-2020
Complete List of Authors:	Bernardo-Castro, Sara; Centro Hospitalar e Universitário de Coimbra EPE, Stroke Unit<br>Donato, Helena; Centro Hospitalar e Universitario de Coimbra EPE, Documentation Service<br>Ferreira, Lino; Center for Neurosciences and Cell Biology, Universidade de Coimbra; Universidade de Coimbra, Faculdade de Medicina<br>Sargento-Freitas, João; Centro Hospitalar e Universitario de Coimbra EPE, Stroke Unit; Universidade de Coimbra, Faculdade de Medicina
Keywords:	Stroke < NEUROLOGY, Magnetic resonance imaging < RADIOLOGY & IMAGING, Neuroradiology < RADIOLOGY & IMAGING, VASCULAR MEDICINE, Computed tomography < RADIOLOGY & IMAGING

SCHOLARONE™
Manuscripts

I, the Submitting Author has the right to grant and does grant on behalf of all authors of the Work (as defined in the below author licence), an exclusive licence and/or a non-exclusive licence for contributions from authors who are: i) UK Crown employees; ii) where BMJ has agreed a CC-BY licence shall apply, and/or iii) in accordance with the terms applicable for US Federal Government officers or employees acting as part of their official duties; on a worldwide, perpetual, irrevocable, royalty-free basis to BMJ Publishing Group Ltd ("BMJ") its licensees and where the relevant Journal is co-owned by BMJ to the co-owners of the Journal, to publish the Work in this journal and any other BMJ products and to exploit all rights, as set out in our licence.

The Submitting Author accepts and understands that any supply made under these terms is made by BMJ to the Submitting Author unless you are acting as an employee on behalf of your employer or a postgraduate student of an affiliated institution which is paying any applicable article publishing charge ("APC") for Open Access articles. Where the Submitting Author wishes to make the Work available on an Open Access basis (and intends to pay the relevant APC), the terms of reuse of such Open Access shall be governed by a Creative Commons licence – details of these licences and which Creative Commons licence will apply to this Work are set out in our licence referred to above.

Other than as permitted in any relevant BMJ Author's Self Archiving Policies, I confirm this Work has not been accepted for publication elsewhere, is not being considered for publication elsewhere and does not duplicate material already published. I confirm all authors consent to publication of this Work and authorise the granting of this licence.

For peer review only

Title: Permeability of the blood brain barrier through the phases of ischemic stroke and relation with clinical outcome: protocol for a systematic review

Authors: Sara Bernardo-Castro[1], Helena Donato[2], Lino Ferreira[3,4], João Sargento-Freitas[1,4]

Affiliations:

1. **Stroke Unit, Centro Hospitalar e Universitário de Coimbra, Coimbra, Portugal**
2. **Documentation Department, Centro Hospitalar e Universitário de Coimbra, Coimbra, Portugal**
3. **Center for Neuroscience and Cell Biology, Universidade de Coimbra, Coimbra, Portugal**
4. **Faculdade de Medicina da Universidade de Coimbra, Coimbra, Portugal**

Corresponding author:

João Sargento-Freitas

Stroke Unit, Centro Hospitalar e Universitário de Coimbra, Coimbra, Portugal

Mail: jsargentof@hotmail.com

Word count: 2193

For peer review only

ABSTRACT

Introduction: Ischemic stroke is the most prevalent type of stroke and is characterized by a myriad of pathological events triggered by a vascular arterial occlusion. Disruption of the blood-brain barrier (BBB) is a key pathological event that may lead to fatal outcomes. However, it seems to follow a multiphasic pattern that has been associated with distinct biological substrates and possibly contrasting outcomes. Addressing the BBB permeability along the different phases of stroke through imaging techniques could lead to a better understanding of the disease and to an improvement on patient selection for specific treatments and development of new therapeutic modalities and delivery methods. This systematic review will aim to comprehensively summarize the existing evidence regarding the evolution of the BBB permeability values during the different phases of an acute ischemic stroke and correlate this event with the clinical outcome of the patient.

Methods and analysis: We will conduct a computerized search on Medline, EMBASE, CENTRAL, Scopus and Web of Science. In addition, grey literature and ClinicalTrials.gov will be scanned. We will include randomized control trials, cohort, cross-sectional and case-controlled studies on humans that quantitatively asses the BBB permeability in stroke. Two independent reviewers will scan the studies, any discrepancies will be solved by consensus or with a third reviewer. Reviewers will extract the data and asses the risk of bias of the selected studies. If possible, data will be combined in a quantitative meta-analysis following the guidelines provided by Cochrane Handbook for Systematic Reviews of Interventions. We will assess cumulative evidence using the Grading of Recommendations, Assessment, Development and Evaluation (GRADE) approach.

Ethics and dissemination: Ethical approval is not needed. All data used for this work is publicly available. The result obtained from this work will be published in a peer-reviewed journal and disseminated in relevant conferences.

PROSPERO registration: CRD42019147314

Strengths and limitations of this study

- To our knowledge this is the first systematic review that will focus on the progression of the BBB permeability during AIS and its clinical consequences.
- This protocol has been developed following Preferred Reporting Items for Systematic Review for systematic review protocols (PRISMA-P).
- This systematic review could help to guide conventional recanalization treatments outside the therapeutic windows and other innovative delivery treatments in later stage of AIS.
- We will include all types of studies, not imposing any restriction on language or year.
- The vast heterogeneity that can arise from the different imaging techniques and outcomes could difficult the performance of a quantitative meta-analysis.

For peer review only

INTRODUCTION

Stroke is the second leading cause of mortality and morbidity worldwide.[1-4] Every year around 14 million people suffer stroke, 5.5 million of which die[1] and another 5 million stay permanently disabled representing a significant concern for public health and society.[2] Acute ischemic strokes (AIS) account for approximately 85% of all strokes,[1,3,4] they restrict blood flow to a specific region of the brain leading to death of the compromised tissue.[5,6] Currently, the only available and effective treatment to limit this situation is recanalization therapy, to restore the normal blood flow,[7] but these therapies can only be given to less than 5% of patients due to the narrow therapeutic window of the disease.[4,7] Treating patients outside this window, could contribute to an additional tissue damage and an increase of the risk of hemorrhagic transformation (HT).[7,8]

During the process of ischemia the blood brain barrier (BBB) undergoes a dysfunction[9,10] that leads to an increase of its permeability,[10] enabling the passage of large molecules, fluids and blood into the brain interstitium.[8,10] This pathological leakage is associated with a worst outcome after AIS[3,8,11] and it is known to persists for days after the stroke[7] following a time-course mediated by complex pathophysiological events with different clinical implications.[12-15] During the first hours after stroke, namely the hyperacute and acute phases, the insult triggers ischemic cell death which leads to a higher risk of HT.[13,16] In a late acute/early subacute stage, the BBB is disrupted due to the secondary ischemic injury which causes inflammatory cell infiltration a tissue scaring.[16,17] In a later subacute stage, the BBB permeability (BBBP) relies on physiological recovery processes such as neoangiogenesis, as demonstrated in both animals[18,19] and humans.[20] Whereas this permeability process is certain to occur, its concrete evolution on humans is not yet certain. A quantitative assessment of the BBB disruption through its permeability could add valuable information in the evaluation of AIS patients. Specific imaging tools known as the brain permeability imaging techniques,[21] are able to measure the BBBP in vivo in a non-invasive manner,[3,11,22] allowing the identification of patients who could benefit from safer and improved recanalization therapies in an extended time window[7-10,21,23-25] as Ryu et al concluded in their systematic review.[26] Nonetheless, although there are important systematic reviews that focus on the implications of imaging and increased permeability on stroke outcome,[27,28] to date there are no systematic reviews, to our knowledge, focusing on the development of the BBBP during the phases of AIS. Very few individual human studies focus on studying this evolution[9,20,29,30] and even though these studies point to a continuous opening of the BBB, they do not offer a clear and collective evidence on the magnitude of this opening in the different phases.

Ideally, this knowledge would help not only in extending the treatment window, but also in the development of future treatment options such as delivery system strategies for neuroprotective or neurorestorative treatments that aim to use the BBB as a therapeutic vehicle or target. Therefore, there is a need to perform a systematic review and meta-analysis on the BBBP dynamics after AIS to gather larger sample sizes of patients and create a concrete understanding of the subject. This systematic review will provide an insight on the evolution of the permeability of the blood brain barrier in patients affected by AIS through the different stages of the stroke and its relevance in the patient outcome and treatment.

For peer review only - http://bmjopen.bmj.com/site/about/guidelines.xhtml

Objective

The main objective of this work is to carry out a systematic review and meta-analysis on the evolution of the permeability values of the blood-brain barrier during the different phases of an acute ischemic stroke and correlate this event with the clinical outcome of the patient.

METHODS

Eligibility criteria

This work will identify randomized control trials, cohort studies (prospective or retrospective), cross-sectional studies and case-controlled studies that asses the BBBP in a quantitative manner using neuroimaging techniques, in patients suffering from AIS. Studies further fulfilling the eligibility criteria shown in Table 1, will be selected for further review. If there is more than one work reporting the same study, the one with the biggest amount of data or the one reporting more relevant data for our propose will be selected. No restriction regarding publication year will be set; therefore, we will be including studies since inception to 31st July 2019. In addition, no language restriction will be applied.

Table 1. Inclusion/exclusion criteria for study selection

Inclusion criteria	Exclusion criteria
Studies on living humans	Non-human studies
Acute ischemic stroke	Lacunar, mild or hemorrhagic stroke
BBBP evaluation through neuroimaging	BBBP evaluation through non-imaging techniques
Studies with a follow up for clinical outcome	BBBP evaluation in other non-AIS diseases
	No primary research
	Reports just defining a study protocol
	Case-report studies
	Studies not reporting time from onset to imaging
	Studies no reporting contralateral permeability values

Information sources

We will conduct a comprehensive computerized literature search strategy to find the studies that will take part in this systematic review. We will search for both, published and unpublished studies in the following data bases: PubMed/MEDLINE, EMBASE, Web of Science, Scopus and Cochrane Central Register of Controlled Trials (CENTRAL). Other electronic platforms such as ClinicalTrials.gov will be scanned to keep-up with ongoing or unpublished clinical trials. In addition to this electronic search, a supplementary search of the grey literature will be conducted with the aim of include all possible existing articles on the subject.

Search strategy

The search strategy will include the following terms and all of its variants in multiple combinations adapted to each one of the data bases regarding its own special requirements as shown in Table 2: ‘stroke’, ‘permeability’, ‘blood brain barrier’, ‘imaging’, ‘neuroimaging’. The search of the grey literature will include a by-hand search of relevant articles in the listed bibliography of the selected studies and important reviews on the subject, conference papers and a google search of the used terms.

For peer review only

Table 2. Retrieval search strategy

PubMed	
**Query**	**Search**
#1	"Stroke"[MeSH Terms] OR "stroke" OR "cerebral stroke" OR "ischemic stroke" OR "acute stroke" OR "acute ischemic stroke" OR "apoplexy" OR "cerebral apoplexy" OR "cerebrovascular accident" OR "acute cerebrovascular accident" OR "brain vascular accident" OR "CVA"
#2	"Blood-Brain Barrier"[MeSH Terms] OR "Blood-Brain Barrier" OR "Blood Brain Barrier" OR "Brain-Blood Barrier" OR "Hemato Encephalic Barrier" OR "Hemato-Encephalic Barrier"
#3	"Permeability"[MeSH Terms] OR "Permeability"
#4	"Diagnostic Imaging"[Mesh] OR "Neuroimaging" [MeSH Terms] OR "Neuroimaging" OR "Brain Imaging" OR "Magnetic Resonance Imaging"[MeSH Terms] OR "Magnetic Resonance Imaging" OR "MRI" OR "MRI scan" OR "Functional MRI" OR "fMRI" OR "Computed Tomography Angiography"[MeSH Terms] OR "Computed Tomography Angiography" OR "Computed Tomography" OR "CT" OR "CT angiography"
#5	Search #1 AND #2 AND #3 AND #4
EMBASE	
#1	'cerebrovascular accident'/exp OR 'brain ischemia'/exp OR 'cerebrovascular accident' OR 'stroke patient' OR 'brain ischemia' OR 'stroke' OR 'acute ischemic stroke' OR 'ischemic stroke' OR 'apoplexy' OR 'cerebral apoplexy' OR 'brain apoplexy'
#2	'blood brain barrier'/exp OR 'blood brain barrier' OR 'hemato encephalic barrier' OR 'hemato-encephalic barrier'
#3	'permeability'/exp OR 'permeability'
#4	'diagnostic imaging'/exp OR 'neuroimaging'/exp OR 'functional magnetic resonance imaging'/exp OR 'nuclear magnetic resonance imaging'/exp OR 'nuclear magnetic resonance'/exp OR 'computer assisted tomography'/exp OR neuroimaging' OR 'nuclear magnetic resonance imaging' OR 'mri' OR 'functional magnetic imaging' OR 'fmri' OR 'computer assisted tomography' OR 'computed tomographic angiography' OR 'ct'
#5	#1 AND #2 AND #3 AND #4
Scopus	
#1	TITLE-ABS-KEY ("stroke") OR TITLE-ABS-KEY ("ischemic stroke") OR TITLE-ABS-KEY ("acute ischemic stroke") OR TITLE-ABS-KEY ("cerebral apoplexy") OR TITLE-ABS-KEY ("cerebrovascular accident") OR TITLE-ABS-KEY ("acute cerebrovascular accident") OR TITLE-ABS-KEY ("brain apoplexy") OR TITLE-ABS-KEY ("CVA")
#2	("blood-brain barrier") OR TITLE-ABS-KEY ("hemato encephalic barrier") OR TITLE-ABS-KEY ("blood brain barrier") OR TITLE-ABS-KEY ("hemato-encephalic barrier")
#3	TITLE-ABS-KEY ("permeability")
#4	TITLE-ABS-KEY ("neuroimaging") OR TITLE-ABS-KEY ("magnetic resonance imaging") OR TITLE-ABS-KEY ("functional magnetic resonance imaging") OR TITLE-ABS-KEY ("MRI") OR TITLE-ABS-KEY ("fMRI") OR TITLE-ABS-KEY ("computed tomography") OR TITLE-ABS-KEY ("computed tomography angiography") OR TITLE-ABS-KEY ("CT"))
CENTRAL	
#1	('blood brain barrier' OR 'hemato encephalic barrier' OR 'hemato-encephalic barrier') AND 'permeability' AND ('cerebrovascular accident' OR 'stroke patient' OR 'brain ischemia' OR 'stroke' OR 'acute ischemic stroke' OR 'ischemic stroke' OR 'apoplexy' OR 'cerebral apoplexy' OR 'brain apoplexy') AND ('neuroimaging' OR 'nuclear magnetic resonance imaging' OR 'mri' OR 'functional magnetic imaging' OR 'fmri' OR 'computer assisted tomography' OR 'computed tomographic angiography' OR 'ct')
Web of Science	
#1	TS=('blood brain barrier' OR 'hemato encephalic barrier' OR 'hemato-encephalic barrier') AND TS=('permeability') AND TS=('cerebrovascular accident' OR 'stroke patient' OR 'brain ischemia' OR 'stroke' OR 'acute ischemic stroke' OR 'ischemic stroke' OR 'apoplexy' OR 'cerebral apoplexy' OR 'brain apoplexy') AND TS=('neuroimaging' OR 'nuclear magnetic resonance imaging' OR 'mri' OR 'functional magnetic imaging' OR 'fmri' OR 'computer assisted tomography' OR 'computed tomographic angiography' OR 'ct')

For peer review only

Data management

All publications arising from the literature search conducted, will be imported to the Mendeley citation software where duplicates will be managed and erased and titles/abstracts of all records will be scanned.

Selection process

Two independent reviewers will conduct the selection process. All records identified in the search stage will be screened by title/abstract and studies clearly not matching the criteria will be discarded. The remaining studies will be full-text reviewed and included or discarded according to the inclusion/exclusion criteria. Any disagreement between the reviewers will be solved by consensus or by a third one if necessary. Reasons for the exclusion of full text records will be recorded. Details on the selection process of the studies will be documented into a flow chart following the Preferred Reporting Items for Systematic Reviews and Meta-Analyses (PRIMSA)[31] as presented in Figure 1.

Data collection process

To ensure that all relevant information is captured, and to minimize the risk of bias, two reviewers will independently extract the information from the studies following the same pilot form. Any disagreement will be solved by consensus. The data extracted will be reviewed and validated by a third reviewer.

Data items

Four main categories of data will be extracted from all studies selected: (1) Features of the study; (2) Patients characteristics; (3) Intervention; (4) Outcome. Among these categories a number of items will be collected as presented in Table 3.

Table 3. Data items to be collected from the selected studies

Features of the study	Patient characteristics	Intervention	Outcome
Title, author	Age, gender	Time form onset to imaging	Permeability values
Study design	Co-morbidities	Imaging characteristics	Final lesion (volume)
Recruitment procedure and duration	NHISS at admission	BBBP assessment characteristics	Follow up (length, number)
Number of participants	Type of stroke	Treatment given	Clinical outcome (NHISS and mRS)
Imaging modality	Vascular territory		Hemorrhagic transformation

Since the main aim of this work is to study the BBBP values in the different phases of stroke, we will form the following groups according to time from onset to imaging reported in each study:

1. Hyperacute stage: 6 hours or less.
2. Acute stage: between 6-48 hours.
3. Subacute stage: between 3-9 days.
4. Chronic stage: 30 or more days.

For peer review only

Outcomes and prioritization

This work has three primary outcomes: (1) the comparison of the quantitative permeability values across time after stroke; (2) The association between the different blood brain barrier permeability values and the functional outcome of acute stroke patients; (3) Association between permeability values and the recanalization treatment given.

When and if possible, the following secondary outcomes will be measured: (1) The association between the different blood brain barrier permeability values and hemorrhagic transformation; (2) Association between any clinical feature/stroke predictor (age, hypertension, diabetes etc.) and the BBB permeability.

Risk of bias in individual studies

With the aim of minimizing bias, the methodological quality of all studies included in the systematic review, will be assessed independently by two reviewers. Since we will be including diverse types of studies, we will use different tools to assess the risk of bias depending of the characteristics of the studies, tuning these tools if necessary. For the RCT we will be using the Cochrane Collaboration's tool for assessing risk of bias in randomized trials.[32] This tool covers seven sources of bias: (1) random sequence generation; (2) allocation concealment; (3) blinding of participants and personnel; (4) blinding of outcome assessment; (5) incomplete outcome data; (6) selective reporting and (7) other bias. For non-randomize trials s we will use the Newcastle-Ottawa Scale (NOS) for assessing the quality of non-randomized studies in meta-analysis.[33] These studies will be assessed based on three perspectives: (1) selection of study groups, (2) Comparability of the groups; (3) Ascertainment of exposure (in case-control studies) or outcome of interest (in cohort studies). Any disagreement between the two reviewers will be solved by consensus or by a third reviewer if necessary.

Data synthesis

This systematic review will include a quantitative meta-analysis if possible. The statistical analysis will be carried out taking into account the guidelines provided by Cochrane Handbook for Systematic Reviews of Interventions.[32] As our main outcome will be presented as continuous data (permeability values), we will use the mean difference (MD) or the standardized mean difference (SMD) and the respective 95% CI to combine the results. We will test the consistency and heterogeneity of the studies with the Higgins I^2 statistic that can also be used to describe heterogeneity among subgroups.[34] Following the direction given by Higgings et al[34] we will consider ≤25% as low heterogeneity, between 25 and 50% as moderate heterogeneity and >75% as high heterogeneity. If the I^2 value is ≤50% (low to moderate heterogeneity) we will use the fixed effect model for data synthesis, if it is greater than 50%, we will use the random effects model. If the heterogeneity values are over 75%, we will search for the possible sources of this high heterogeneity, including reviewing the methodological processes of the selected studies, and the search of outliers or influential cases that may distort the results of the analysis. If we consider that a quantitative meta-analysis is not feasible, we will conduct a narrative description.[35]

For peer review only

Subgroup analysis

When possible, the following subgroups will be made:

- Subgroups according to the imaging technique used with the aim of reduce possible heterogeneity arising from this methodological variety.
- Subgroups according to the treatment received.
- Subgroups according to the presence/absence of HT.
- Subgroups according to the mRS 90 days value: (1) mRS 0-1; (2) mRS 2-5

We will compare the permeability values among the subgroups and, if possible, correlate these with the different features/predictors of stroke.

Meta-bias(es)

To assess the publication bias, we will conduct a funnel plot following the recommendation of the Cochrane Handbook for Systematic Reviews of Interventions.[32]

Confidence in cumulative evidence

The strength of the body evidence will be assessed using the Grading Recommendations Assessment, Development and Evaluation (GRADE).[32]

Patient and public involvement

This is a protocol for a systematic review that will be based on previously published data, therefore no participant recruitment will take place. The involvement of participants on the recruitment and dissemination of results is not applicable.

ETHICS AND DISSEMINATION

Due to this work will be based in data that are public and already published, an ethical approval would not be necessary. The result obtained from this work will be published in a peer-reviewed journal and disseminated in relevant conferences. If any amendments are needed due to any deviations from this protocol in the execution of the study, these amendments will be recorded and noted in the publication.

Authors' contributions: JSF and SBC formulated the idea for this systematic review. SBC drafted the protocol guided by JSF and HD. JSF, HD and LF reviewed all manuscript versions.

Funding: This work is framed within the NANOSTEM project. This project has received funding from the European Union's Horizon 2020 research and innovation program under grant agreement number 764958.

Competing interests' statement: None declared.

For peer review only

REFERENCES

1 GBD 2016 Stroke Collaborators. Global, regional, and national burden of stroke, 1990-2016: a systematic analysis for the Global Burden of Disease Study 2016. *Lancet Neurol* 2019;18:439-458.

2 Rajsic S, Gothe H, Borba HH, et al. Economic burden of stroke: a systematic review on post stroke care. *Eur J Health Econ* 2019;20:107-134.

3 Hoffmann A, Zhu G, Wintermark M. Advanced neuroimaging in stroke patients: Prediction of tissue fate and hemorrhagic transformation. *Expert Rev Cardiovasc Ther* 2012;10:515–524.

4 Matta R, Gonzalez AL. Stroke repair Via biomimicry of the subventricular zone. *Front Mater* 2018;5:15.

5 Wey HY, Desai VR, Duong TQ. A review of current imaging methods used in stroke research. *Neurol Res* 2013;35:1092–1102.

6 Barber PA. Magnetic resonance imaging of ischemia viability thresholds and the neurovascular unit. *Sensors* 2013;13:6981–7003.

7 Kassner A, Merali Z. Assessment of Blood-Brain Barrier Disruption in Stroke. *Stroke* 2015;46:3310–3315.

8 Avsenik J, Bisdas S, Popovic KS. Blood-brain barrier permeability imaging using perfusion computed tomography. *Radiol Oncol* 2015;49:107–114.

9 Villringer K, Sanz Cuesta BE, Ostwaldt AC, et al. DCE-MRI blood-brain barrier assessment in acute ischemic stroke. *Neurology* 2017;88:433-440.

10 Jiang X, Andjelkovic AV, Zhu L, et al. Blood-brain barrier dysfunction and recovery after ischemic stroke. *Prog Neurobiol* 2018;163-164:144-171.

11 Jiang Q, Ewing JR, Chopp M. MRI of Blood-Brain Barrier Permeability in cerebral ischemia. *Transl Stroke Res* 2012;3:56–64.

12 Yang Y, Rosenberg GA. Blood-brain barrier breakdown in acute and chronic cerebrovascular disease. *Stroke* 2011;42:3323-8.

13 Bernhardt J, Hayward KS, Kwakkel G, et al. Agreed Definitions and a Shared Vision for New Standards in Stroke Recovery Research: The Stroke Recovery and Rehabilitation Roundtable Taskforce. *Neurorehabil Neural Repair* 2017;31:793-799.

14 De Simoni MG, Milia P, Barba M, et al. The inflammatory response in cerebral ischemia: Focus on cytokines in stroke patients. *Clin Exp Hypertens* 2002;24:535–542.

15 Mehta SL, Manhas N, Raghubir R. Molecular targets in cerebral ischemia for developing novel therapeutics. *Brain Res Rev* 2007;54:34–66.

16 Carmichael ST. The 3 Rs of Stroke Biology: Radial, Relayed, and Regenerative. *Neurotherapeutics* 2016;13:348–359.

17 Heiss WD. The ischemic penumbra: How does tissue injury evolve? *Ann N Y Acad Sci* 2012;1268:26–34.

18 Chopp M, Zhang ZG, Jiang Q. Neurogenesis, angiogenesis, and MRI indices of functional recovery from stroke. *Stroke* 2007;38(Suppl 2):827–831.

19 Ding G, Jiang Q, Li L, et al. Angiogenesis detected after embolic stroke in rat brain using magnetic resonance T2*WI. *Stroke* 2008;39:1563-8.

20 Sargento-Freitas J, Aday S, Nunes C et al. Endothelial progenitor cells enhance blood-brain barrier permeability in subacute stroke. *Neurology* 2018;90:e127–e134.

21 Chen H, Zhu G, Liu N, et al. Applications and development of permeability imaging in ischemic stroke. *Exp Ther Med* 2018;16:2203–2207.

For peer review only

22 Mikulis DJ. Functional cerebrovascular imaging in brain ischemia: Permeability, reactivity, and functional MR imaging. *Neuroimaging Clin N Am* 2005;15:667–680.

23 Harring M, Leonardo J, Gibbons K, et al. CT Perfusion Cerebral Blood Flow Imaging in Neurological Critical Care. *Neurocrit Care* 2005;2:306–312.

24 Kassner A, Mandell DM, Mikulis DJ. Measuring Permeability in Acute Ischemic Stroke. *Neuroimaging Clin N Am* 2001;21:315–325.

25 Jaillard A, Cronu C, Durieux A, et al. Hemorrhagic Transformation in Acute Ischemic Stroke The MAST-E Study. MAST-E Group. *Stroke* 1999;30:1326–1332.

26 Ryu WHA, Avery MB, Dharampal N, et al. Utility of perfusion imaging in acute stroke treatment: a systematic review and meta-analysis. *J Neurointerv Surg* 2017;9:1012-1016.

27 Suh CH, Jung SC, Cho SJ, et al. MRI for prediction of hemorrhagic transformation in acute ischemic stroke: a systematic review and meta-analysis. *Acta Radiol* 2019;18:284185119887593

28 Suh CH, Jung SC, Cho SJ, et al. Perfusion CT for prediction of hemorrhagic transformation in acute ischemic stroke: a systematic review and meta-analysis. *Eur Radiol* 2019;29:4077-4087.

29 Merali Z, Huang K, Mikulis D, et al. Evolution of blood-brain-barrier permeability after acute ischemic stroke. *PLoS One* 2017;12:1–11.

30 Liu HS, Chung HW, Chou MC, et al. Effects of microvascular permeability changes on contrast-enhanced t1 and pharmacokinetic MR imagings after ischemia. *Stroke* 2013;44:1872–1877.

31 Liberati A, Altman DG, Tetzlaff J, et al. The PRISMA statement for reporting systematic reviews and meta-analyses of studies that evaluate health care interventions: explanation and elaboration. *PLoS Med* 2009 21;6:e1000100.

32 Higgins JPT, Green S. Cochrane Handbook for Systematic Reviews of Interventions Version 5.1.0 [updated March 2011]. The Cochrane Collaboration 2011. Available at: www.cochrane-handbook.org.

33 Wells G, Shea B, O'Connell D et al. The Newcastle-Ottawa Scale (NOS) for assessing the quality of nonrandomised studies in meta-analyses. 2008. Available at: http://www.ohri.ca/programs/clinical_epidemiology/oxford.asp. (Accessed: 5th August 2019)

34 Higgings J, Thompson S, Jonathan D et al. Measuring inconsistency in meta-analyses. *BMJ* 2003:327;557–560.

35 Thanvi BR, Treadwell S, Robinson T. Haemorrhagic transformation in acute ischaemic stroke following thrombolysis therapy: classification, pathogenesis and risk factors. *Postgrad Med J* 2008;84:361–367.

Legends

Figure 1. Flow-chart diagram presenting the selection process for the studies.

For peer review only

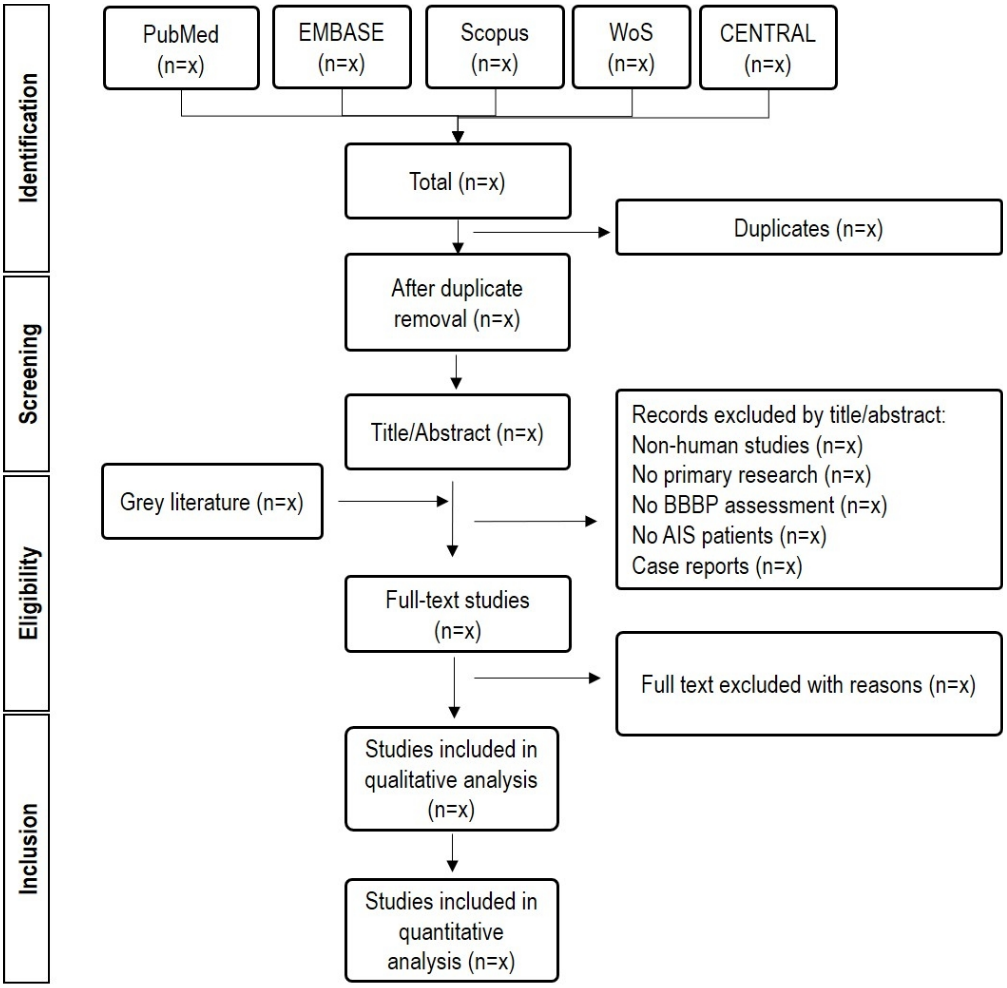

Figure 1. Flow-chart diagram presenting the selection process for the studies.

178x175mm (150 x 150 DPI)

PRISMA-P (Preferred Reporting Items for Systematic review and Meta-Analysis Protocols) 2015 checklist: recommended items to address in a systematic review protocol*

Section and topic	Item No	Checklist item
**ADMINISTRATIVE INFORMATION**		
Title:		
Identification	1a	Identify the report as a protocol of a systematic review
Update	1b	If the protocol is for an update of a previous systematic review, identify as such
Registration	2	If registered, provide the name of the registry (such as PROSPERO) and registration number
Authors:		
Contact	3a	Provide name, institutional affiliation, e-mail address of all protocol authors; provide physical mailing address of corresponding author
Contributions	3b	Describe contributions of protocol authors and identify the guarantor of the review
Amendments	4	If the protocol represents an amendment of a previously completed or published protocol, identify as such and list changes; otherwise, state plan for documenting important protocol amendments
Support:		
Sources	5a	Indicate sources of financial or other support for the review
Sponsor	5b	Provide name for the review funder and/or sponsor
Role of sponsor or funder	5c	Describe roles of funder(s), sponsor(s), and/or institution(s), if any, in developing the protocol
**INTRODUCTION**		
Rationale	6	Describe the rationale for the review in the context of what is already known
Objectives	7	Provide an explicit statement of the question(s) the review will address with reference to participants, interventions, comparators, and outcomes (PICO)
**METHODS**		
Eligibility criteria	8	Specify the study characteristics (such as PICO, study design, setting, time frame) and report characteristics (such as years considered, language, publication status) to be used as criteria for eligibility for the review
Information sources	9	Describe all intended information sources (such as electronic databases, contact with study authors, trial registers or other grey literature sources) with planned dates of coverage
Search strategy	10	Present draft of search strategy to be used for at least one electronic database, including planned limits, such that it could be repeated
Study records:		
Data management	11a	Describe the mechanism(s) that will be used to manage records and data throughout the review

For peer review only

Selection process	11b	State the process that will be used for selecting studies (such as two independent reviewers) through each phase of the review (that is, screening, eligibility and inclusion in meta-analysis)
Data collection process	11c	Describe planned method of extracting data from reports (such as piloting forms, done independently, in duplicate), any processes for obtaining and confirming data from investigators
Data items	12	List and define all variables for which data will be sought (such as PICO items, funding sources), any pre-planned data assumptions and simplifications
Outcomes and prioritization	13	List and define all outcomes for which data will be sought, including prioritization of main and additional outcomes, with rationale
Risk of bias in individual studies	14	Describe anticipated methods for assessing risk of bias of individual studies, including whether this will be done at the outcome or study level, or both; state how this information will be used in data synthesis
Data synthesis	15a	Describe criteria under which study data will be quantitatively synthesised
	15b	If data are appropriate for quantitative synthesis, describe planned summary measures, methods of handling data and methods of combining data from studies, including any planned exploration of consistency (such as I^2, Kendall's τ)
	15c	Describe any proposed additional analyses (such as sensitivity or subgroup analyses, meta-regression)
	15d	If quantitative synthesis is not appropriate, describe the type of summary planned
Meta-bias(es)	16	Specify any planned assessment of meta-bias(es) (such as publication bias across studies, selective reporting within studies)
Confidence in cumulative evidence	17	Describe how the strength of the body of evidence will be assessed (such as GRADE)

*** It is strongly recommended that this checklist be read in conjunction with the PRISMA-P Explanation and Elaboration (cite when available) for important clarification on the items. Amendments to a review protocol should be tracked and dated. The copyright for PRISMA-P (including checklist) is held by the PRISMA-P Group and is distributed under a Creative Commons Attribution Licence 4.0.**

From: Shamseer L, Moher D, Clarke M, Ghersi D, Liberati A, Petticrew M, Shekelle P, Stewart L, PRISMA-P Group. Preferred reporting items for systematic review and meta-analysis protocols (PRISMA-P) 2015: elaboration and explanation. BMJ. 2015 Jan 2;349(jan02 1):g7647.

For peer review only

BMJ Open

Permeability of the blood brain barrier through the phases of ischemic stroke and relation with clinical outcome: protocol for a systematic review

Journal:	*BMJ Open*
Manuscript ID	bmjopen-2020-039280.R1
Article Type:	Protocol
Date Submitted by the Author:	10-Jun-2020
Complete List of Authors:	Bernardo-Castro, Sara; Centro Hospitalar e Universitário de Coimbra EPE, Stroke Unit<br>Donato, Helena; Centro Hospitalar e Universitario de Coimbra EPE, Documentation Service<br>Ferreira, Lino; Center for Neurosciences and Cell Biology, Universidade de Coimbra; Universidade de Coimbra, Faculdade de Medicina<br>Sargento-Freitas, João; Centro Hospitalar e Universitario de Coimbra EPE, Stroke Unit; Universidade de Coimbra, Faculdade de Medicina
**Primary Subject Heading**:	Neurology
Secondary Subject Heading:	Radiology and imaging
Keywords:	Stroke < NEUROLOGY, Magnetic resonance imaging < RADIOLOGY & IMAGING, Neuroradiology < RADIOLOGY & IMAGING, VASCULAR MEDICINE, Computed tomography < RADIOLOGY & IMAGING

SCHOLARONE™
Manuscripts

BMJ

I, the Submitting Author has the right to grant and does grant on behalf of all authors of the Work (as defined in the below author licence), an exclusive licence and/or a non-exclusive licence for contributions from authors who are: i) UK Crown employees; ii) where BMJ has agreed a CC-BY licence shall apply, and/or iii) in accordance with the terms applicable for US Federal Government officers or employees acting as part of their official duties; on a worldwide, perpetual, irrevocable, royalty-free basis to BMJ Publishing Group Ltd ("BMJ") its licensees and where the relevant Journal is co-owned by BMJ to the co-owners of the Journal, to publish the Work in this journal and any other BMJ products and to exploit all rights, as set out in our licence.

The Submitting Author accepts and understands that any supply made under these terms is made by BMJ to the Submitting Author unless you are acting as an employee on behalf of your employer or a postgraduate student of an affiliated institution which is paying any applicable article publishing charge ("APC") for Open Access articles. Where the Submitting Author wishes to make the Work available on an Open Access basis (and intends to pay the relevant APC), the terms of reuse of such Open Access shall be governed by a Creative Commons licence – details of these licences and which Creative Commons licence will apply to this Work are set out in our licence referred to above.

Other than as permitted in any relevant BMJ Author's Self Archiving Policies, I confirm this Work has not been accepted for publication elsewhere, is not being considered for publication elsewhere and does not duplicate material already published. I confirm all authors consent to publication of this Work and authorise the granting of this licence.

For peer review only

Title: Permeability of the blood brain barrier through the phases of ischemic stroke and relation with clinical outcome: protocol for a systematic review

Authors: Sara Bernardo-Castro[1], Helena Donato[2], Lino Ferreira[3,4], João Sargento-Freitas[1,4]

Affiliations:

1. **Stroke Unit, Centro Hospitalar e Universitário de Coimbra, Coimbra, Portugal**
2. **Documentation Department, Centro Hospitalar e Universitário de Coimbra, Coimbra, Portugal**
3. **Center for Neuroscience and Cell Biology, Universidade de Coimbra, Coimbra, Portugal**
4. **Faculdade de Medicina da Universidade de Coimbra, Coimbra, Portugal**

Corresponding author:

João Sargento-Freitas

Stroke Unit, Centro Hospitalar e Universitário de Coimbra, Coimbra, Portugal

Mail: jsargentof@hotmail.com

Word count: 2586

For peer review only

ABSTRACT

Introduction: Ischemic stroke is the most prevalent type of stroke and is characterized by a myriad of pathological events triggered by a vascular arterial occlusion. Disruption of the blood-brain barrier (BBB) is a key pathological event that may lead to fatal outcomes. However, it seems to follow a multiphasic pattern that has been associated with distinct biological substrates and possibly contrasting outcomes. Addressing the BBB permeability along the different phases of stroke through imaging techniques could lead to a better understanding of the disease, improved patient selection for specific treatments and development of new therapeutic modalities and delivery methods. This systematic review will aim to comprehensively summarize the existing evidence regarding the evolution of the BBB permeability values during the different phases of an acute ischemic stroke and correlate this event with the clinical outcome of the patient.

Methods and analysis: We will conduct a computerized search on Medline, EMBASE, CENTRAL, Scopus and Web of Science. In addition, grey literature and ClinicalTrials.gov will be scanned. We will include randomized control trials, cohort, cross-sectional and case-controlled studies on humans that quantitatively assess the BBB permeability in stroke. Retrieved studies will be independently reviewed by two authors and any discrepancies will be resolved by consensus or with a third reviewer. Reviewers will extract the data and assess the risk of bias of the selected studies. If possible, data will be combined in a quantitative meta-analysis following the guidelines provided by Cochrane Handbook for Systematic Reviews of Interventions. We will assess cumulative evidence using the Grading of Recommendations, Assessment, Development and Evaluation (GRADE) approach.

Ethics and dissemination: Ethical approval is not needed. All data used for this work is publicly available. The result obtained from this work will be published in a peer-reviewed journal and disseminated in relevant conferences.

PROSPERO registration: CRD42019147314

Strengths and limitations of this study

- To our knowledge this is the first systematic review that will focus on the progression of the BBB permeability during AIS and its clinical consequences.
- This protocol has been developed following Preferred Reporting Items for Systematic Review for systematic review protocols (PRISMA-P).
- This systematic review could help to guide conventional recanalization treatments outside the therapeutic windows and other innovative delivery treatments in later stage of AIS.
- We will include all types of studies, not imposing any restriction on language or year.
- The vast heterogeneity that can arise from the use of different BBBP imaging techniques, the pharmacokinetic models used and the different permeability parameters yield from each technique, may prevent a quantitative meta-analysis from being conducted.

For peer review only

INTRODUCTION

Stroke is the second leading cause of mortality and morbidity worldwide.[1–4] Every year around 14 million people suffer a stroke, 5.5 million of which die[1] and another 5 million stay permanently disabled, representing a significant concern for public health and society.[2] Acute ischemic strokes (AIS) account for approximately 85% of all strokes,[1,3,4] they restrict blood flow to a specific region of the brain leading to death of the compromised tissue.[5,6] Currently, the only available and effective treatment to limit this situation is recanalization therapy, to restore the normal blood flow,[7] but these therapies can only be given to less than 5% of patients due to their narrow therapeutic window.[4,7] Treating patients outside this window, could contribute to additional tissue damage and increase in the risk of hemorrhagic transformation (HT).[7,8]

The blood-brain barrier (BBB) is a dynamic physiological structure that constitutes an interface between the vasculature system and the neural tissues. It regulates the transport of substances in a bi-directional way[9] and protects the central nervous system (CNS) from unwanted compounds, playing a crucial role in maintaining its homeostasis.[9,10] During the process of ischemia the BBB undergoes a dysfunction[9,11] that leads to an increase of its permeability,[10] enabling the passage of large molecules, fluids and blood into the brain interstitium.[8,9] This pathological leakage is associated with a worst outcome after AIS[3,8,12] and is known to persists for several days[7] following a time-course mediated by complex pathophysiological events with different clinical implications.[13–15] During the first hours after stroke, namely the hyperacute and acute phases, the insult triggers ischemic cell death which leads to a higher risk of HT.[16,17] In a late acute/early subacute stage, the BBB is disrupted due to the secondary ischemic injury which causes inflammatory cell infiltration and tissue scaring and a further BBB permeability (BBBP) increase.[17,18] In a later subacute stage, the BBBP relies on physiological recovery processes such as neoangiogenesis, as demonstrated in both animals[19,20] and humans.[21] Whereas the existence of this permeability process is unequivocal, its concrete evolution is not yet certain.

Several longitudinal animal studies have tried to explain this event. Some studies point to an “open-close-open” biphasic pattern in which the BBB has increased permeability at a first stage followed by a return to normal values and a second permeability increase.[22–24] Nonetheless these studies show differences on the open/close times and more recent literature points to a more continuous opening of the BBB with biphasic permeability peaks but without total closing [25–28]. A first BBBP increase has been shown to appear as soon as 3/6 hours after occlusion, followed by a decrease but not a total recovery, and a second peak at the early subacute stage [26,27,29]. Studies extending the BBBP quantification time-points, have reported a further increased permeability up to one[27,29], three[27] and even five weeks[28,30] after occlusion, suggesting that the BBB could remain open until months after the onset of stroke.

Very few human studies have focused on studying this evolution[11,21,31,32] and even though these studies point to a continuous opening of the BBB, they do not offer a clear and collective evidence on the magnitude of this opening in the different phases. A quantitative assessment of the BBB disruption through its permeability could add valuable information in the evaluation of AIS patients.

Three main imaging techniques are used to evaluate BBBP: computed tomography (CT), magnetic resonance imaging (MRI) and positron emission tomography (PET).[33] Nonetheless,

For peer review only

due to the limited availability of PET, the "permeability imaging" term is used mainly for MRI and CT.[7] These specific imaging tools are able to measure the BBBP in vivo in a non-invasive manner.[3,9,11,34–37] In short, these imaging modalities quantify the rate and amount at which a specific contrast agent leaves the blood stream and enters the brain parenchyma[12,34,38] using mathematical models able to describe the physiological characteristics of the BBB such as vessel permeability, vessel surface area product and tissue volume fraction.[11,39,40] In clinical practice this information has been used as a diagnostic tool for differential diagnosis, and also to support decisions for safer and improved recanalization therapies for stroke patients in an extended time window.[7–9,11,38,41–43]

Nonetheless, although there are important systematic reviews that focus on the implications of imaging and increased permeability on stroke outcome,[44,45] and on the utility of perfusion imaging in determining treatment eligibility in patients with acute stroke,[46] to date there are no systematic reviews, to our knowledge, focusing on the development of the BBBP during the phases of AIS.

Ideally, this knowledge would help not only in extending the treatment window, but also in the development of future treatment options such as delivery system strategies for neuroprotective or neurorestorative treatments that aim to use the BBB as a therapeutic vehicle or target. Therefore, there is a need to perform a systematic review and meta-analysis on the BBBP dynamics after AIS to gather larger sample sizes of patients and create a concrete understanding of the subject. This systematic review will provide an insight on the evolution of the permeability of the blood brain barrier in patients affected by AIS through the different stages of the stroke and its relevance in the patient outcome and treatment.

Objective

The main objective of this work is to carry out a systematic review and meta-analysis on the blood-brain barrier permeability during the different phases of an acute ischemic stroke with the aim of assessing its evolution through time and its correlation with clinical outcome.

METHODS

Eligibility criteria

This work will identify randomized controlled trials, cohort studies (prospective or retrospective), cross-sectional studies and case-controlled studies that quantify BBBP in patients suffering from AIS. Studies fulfilling the eligibility criteria shown in Table 1 will be selected for further review. If more than one article reports the same study, the article with the largest sample size or reporting more relevant data for our specific aim will be selected. No restriction regarding publication year will be set; therefore, we will be including studies since inception to 31st July 2019. In addition, no language restriction will be applied. If a study in a non-understandable language is obtained, we will consider its suitability for our study by its English abstract and if the information is interesting enough to be included, the paper will be sent to a professional translator.

For peer review only

Table 1. Inclusion/exclusion criteria for study selection

**Inclusion criteria**	**Exclusion criteria**
Studies on living humans	Non-human studies
Acute ischemic stroke	Lacunar strokes (subcortical ischemic lesion with a diameter under 15mm in CT or 20mm in MRI)
BBBP evaluation through neuroimaging	Mild stroke (NIHSS below 6)
Studies with a follow up for clinical outcome	Hemorrhagic stroke
	BBBP evaluation through non-imaging techniques
	BBBP evaluation in other non-AIS diseases
	No primary research
	Reports just defining a study protocol
	Case-report studies
	Studies not reporting time from onset to imaging
	Studies no reporting contralateral permeability values

For peer review only

Information sources

We will conduct a comprehensive computerized literature search strategy to find the studies that will take part in this systematic review. We will search for both published and unpublished studies in the following data bases: PubMed/MEDLINE, EMBASE, Web of Science, Scopus and Cochrane Central Register of Controlled Trials (CENTRAL). Other electronic platforms such as ClinicalTrials.gov will be scanned to keep-up with ongoing or unpublished clinical trials. If any relevant unpublished trial is found, the corresponding author listed will be contacted to obtain the required information. If no response is given or, if the author decides not to share the data, this will be listed as the reason for exclusion of said trial. In addition to this electronic search, a supplementary search of the grey literature will be conducted with the aim of include all possible existing articles on the subject. No pre-prints will be included on the study.

Search strategy

The search strategy will include the following terms and all of its variants in multiple combinations adapted to each one of the data bases regarding its own special requirements as shown in Table 2: 'stroke', 'permeability', 'blood brain barrier', 'imaging', 'neuroimaging'. The search of the grey literature will include a by-hand search of relevant articles in the listed bibliography of the selected studies and important reviews on the subject, conference papers and a google search of the used terms.

Table 2. Retrieval search strategy

PubMed	
**Query**	**Search**
#1	"Stroke"[MeSH Terms] OR "stroke" OR "cerebral stroke" OR "ischemic stroke" OR "acute stroke" OR "acute ischemic stroke" OR "apoplexy" OR "cerebral apoplexy" OR "cerebrovascular accident" OR "acute cerebrovascular accident" OR "brain vascular accident" OR "CVA"
#2	"Blood-Brain Barrier"[MeSH Terms] OR "Blood-Brain Barrier" OR "Blood Brain Barrier" OR "Brain-Blood Barrier" OR "Hemato Encephalic Barrier" OR "Hemato-Encephalic Barrier"
#3	"Permeability"[MeSH Terms] OR "Permeability" OR "Leakage"
#4	"Diagnostic Imaging"[Mesh] OR "Neuroimaging" [MeSH Terms] OR "Neuroimaging" OR "Brain Imaging" OR "Magnetic Resonance Imaging"[MeSH Terms] OR "Magnetic Resonance Imaging" OR "MRI" OR "MRI scan" OR "Functional MRI" OR "fMRI" OR "Computed Tomography Angiography"[MeSH Terms] OR "Computed Tomography Angiography" OR "Computed Tomography" OR "CT" OR "CT angiography" OR " dynamic contrast enhanced MRI" OR "dynamic susceptibility contrast MRI" OR "computed tomography perfusion"

#5	Search #1 AND #2 AND #3 AND #4
EMBASE	
#1	'cerebrovascular accident'/exp OR 'brain ischemia'/exp OR 'cerebrovascular accident' OR 'stroke patient' OR 'brain ischemia' OR 'stroke' OR 'acute ischemic stroke' OR 'ischemic stroke' OR 'apoplexy' OR 'cerebral apoplexy' OR 'brain apoplexy'
#2	'blood brain barrier'/exp OR 'blood brain barrier' OR 'hemato encephalic barrier' OR 'hemato-encephalic barrier'
#3	'permeability'/exp OR 'permeability' OR 'leakage'
#4	'diagnostic imaging'/exp OR 'neuroimaging'/exp OR 'functional magnetic resonance imaging'/exp OR 'nuclear magnetic resonance imaging'/exp OR 'nuclear magnetic resonance'/exp OR 'computer assisted tomography'/exp OR neuroimaging' OR 'nuclear magnetic resonance imaging' OR 'mri' OR 'functional magnetic imaging' OR 'fmri' OR 'computer assisted tomography' OR 'computed tomographic angiography' OR 'ct' OR 'dynamic contrast enhanced MRI' OR 'dynamic susceptibility contrast MRI' OR 'computed tomography perfusion'
#5	#1 AND #2 AND #3 AND #4
Scopus	
#1	TITLE-ABS-KEY ("stroke") OR TITLE-ABS-KEY ("ischemic stroke") OR TITLE-ABS-KEY ("acute ischemic stroke") OR TITLE-ABS-KEY ("cerebral apoplexy") OR TITLE-ABS-KEY ("cerebrovascular accident") OR TITLE-ABS-KEY ("acute cerebrovascular accident") OR TITLE-ABS-KEY ("brain apoplexy") OR TITLE-ABS-KEY ("CVA")
#2	("blood-brain barrier") OR TITLE-ABS-KEY ("hemato encephalic barrier") OR TITLE-ABS-KEY ("blood brain barrier") OR TITLE-ABS-KEY ("hemato-encephalic barrier")
#3	TITLE-ABS-KEY ("permeability") OR TITLE-ABS-KEY ("leakage")
#4	TITLE-ABS-KEY ("neuroimaging") OR TITLE-ABS-KEY ("magnetic resonance imaging") OR TITLE-ABS-KEY ("functional magnetic resonance imaging") OR TITLE-ABS-KEY ("MRI") OR TITLE-ABS-KEY ("fMRI") OR TITLE-ABS-KEY ("computed tomography") OR TITLE-ABS-KEY ("computed tomography angiography") OR TITLE-ABS-KEY ("CT") OR TITLE-ABS-KEY ("dynamic contrast enhanced MRI") OR TITLE-ABS-KEY ("dynamic susceptibility contrast MRI") OR TITLE-ABS-KEY ("computed tomography perfusion"))
CENTRAL	
#1	('blood brain barrier' OR 'hemato encephalic barrier' OR 'hemato-encephalic barrier') AND ('permeability' OR 'leakage') AND ('cerebrovascular accident' OR 'stroke patient' OR 'brain ischemia' OR 'stroke' OR 'acute ischemic stroke' OR 'ischemic stroke' OR 'apoplexy' OR 'cerebral apoplexy' OR 'brain apoplexy') AND ('neuroimaging' OR 'nuclear magnetic resonance imaging' OR 'mri' OR 'functional magnetic imaging' OR 'fmri' OR 'computer assisted tomography' OR 'computed tomographic angiography' OR 'ct' OR 'dynamic contrast enhanced MRI' OR 'dynamic susceptibility contrast MRI' OR 'computed tomography perfusion')
Web of Science	
#1	TS=('blood brain barrier' OR 'hemato encephalic barrier' OR 'hemato-encephalic barrier') AND TS=('permeability' OR 'leakage') AND TS=('cerebrovascular accident' OR 'stroke patient' OR 'brain ischemia' OR 'stroke' OR 'acute ischemic stroke' OR 'ischemic stroke' OR 'apoplexy' OR 'cerebral apoplexy' OR 'brain apoplexy') AND TS=('neuroimaging' OR 'nuclear magnetic resonance imaging' OR 'mri' OR 'functional magnetic imaging' OR 'fmri' OR 'computer assisted tomography' OR 'computed tomographic angiography' OR 'ct' OR 'dynamic contrast enhanced MRI' OR 'dynamic susceptibility contrast MRI' OR 'computed tomography perfusion')

For peer review only

Data management

All publications arising from the literature search conducted, will be imported to the Mendeley citation software where duplicates will be managed and erased and titles/abstracts of all records will be scanned.

Selection process

Two independent reviewers will conduct the selection process. All records identified in the search stage will be screened by title/abstract and studies clearly not matching the criteria will be discarded. The remaining studies will be full-text reviewed and included or discarded according to the inclusion/exclusion criteria. Any disagreement between the reviewers will be resolved by consensus or by a third one if necessary. Reasons for the exclusion of full text records

will be recorded. Details on the selection process of the studies will be documented into a flow chart following the Preferred Reporting Items for Systematic Reviews and Meta-Analyses (PRIMSA)[47] as presented in Figure 1.

Data collection process

To ensure that all relevant information is captured, and to minimize the risk of bias, two reviewers will independently extract the information from the studies following the same pilot form. Any disagreement will be resolved by consensus. The data extracted will be reviewed and validated by a third reviewer.

Data items

Four main categories of data will be extracted from all studies selected: (1) Features of the study; (2) Patients characteristics; (3) Intervention; (4) Outcome. Among these categories a number of items will be collected as presented in Table 3.

Table 3. Data items to be collected from the selected studies

Features of the study	Patient characteristics	Intervention	Outcome
Title, author	Age, gender	Time form onset to imaging	Permeability values
Study design	Co-morbidities	Imaging characteristics	Final lesion (volume)
Recruitment procedure and duration	NHISS at admission	BBBP assessment characteristics	Follow up (length, number)
Number of participants	Stroke etiology (TOAST classification)	Reperfusion treatment given	Clinical outcome (NHISS and mRS)
Imaging modality	Vascular territory		Hemorrhagic transformation

Since the main aim of this work is to study the BBBP values in the different phases of stroke, we will form the following groups according to time from onset to imaging reported in each study:

1. Hyperacute stage: 6 hours or less.
2. Acute stage: between 6-48 hours.
3. Subacute stage: between 3-9 days.
4. Chronic stage: 30 or more days.

For any study reporting more than one BBBP measurement, each of the measurements will be considered as an independent study and will be placed in the corresponding group according to the time-points established above. These values will be identified as author, year followed by the name of the corresponding stage.

Outcomes and prioritization

This work has three primary outcomes: (1) the comparison of the quantitative permeability values across time after stroke; (2) The association between the different blood brain barrier permeability values and the functional outcome of acute stroke patients; (3) Association between permeability values and the recanalization treatment given.

When and if possible, the following secondary outcomes will be measured: (1) The association between the different blood brain barrier permeability values and hemorrhagic transformation;

For peer review only

(2) Association between any clinical feature/stroke predictor (age, hypertension, diabetes etc.) and the BBB permeability.

Risk of bias in individual studies

With the aim of minimizing bias, the methodological quality of all studies included in the systematic review, will be assessed independently by two reviewers. Since we will be including diverse types of studies, we will use different tools to assess the risk of bias depending of the characteristics of the studies, tuning these tools if necessary. For the RCT we will be using the Cochrane Collaboration's tool for assessing risk of bias in randomized trials.[48] This tool covers seven sources of bias: (1) random sequence generation; (2) allocation concealment; (3) blinding of participants and personnel; (4) blinding of outcome assessment; (5) incomplete outcome data; (6) selective reporting and (7) other bias. For non-randomize trials we will use the Newcastle-Ottawa Scale (NOS) for assessing the quality of non-randomized studies in meta-analysis.[49] These studies will be assessed based on three perspectives: (1) selection of study groups, (2) Comparability of the groups; (3) Ascertainment of exposure (in case-control studies) or outcome of interest (in cohort studies). Any disagreement between the two reviewers will be resolved by consensus or by a third reviewer if necessary.

Data synthesis

This systematic review will include a quantitative meta-analysis if possible. The statistical analysis will be carried out taking into account the guidelines provided by Cochrane Handbook for Systematic Reviews of Interventions.[48] As our main outcome will be presented as continuous data (permeability values), we will use the mean difference (MD) or the standardized mean difference (SMD) and the respective 95% CI to combine the results. We will test the consistency and heterogeneity of the studies with the Higgins I^2 statistic that can also be used to describe heterogeneity among subgroups.[50] Following the direction given by Higgings et al[50] we will consider ≤25% as low heterogeneity, between 25 and 50% as moderate heterogeneity and >75% as high heterogeneity. If the I^2 value is ≤50% (low to moderate heterogeneity) we will use the fixed effect model for data synthesis, if it is greater than 50%, we will use the random effects model. If the heterogeneity values are over 75%, we will search for the possible sources of this high heterogeneity, including reviewing the methodological processes of the selected studies, and search for outliers or influential cases that may distort the results of the analysis. Any possible outlier or influential case, as well as studies presenting with poor methodological quality and/or a high or critical risk of bias, will be excluded in a further sensitivity analysis.

If we are not able to collect the appropriate outcome information or not enough studies are retrieved for the different stages, we will consider that a quantitative meta-analysis is not feasible and we will conduct a narrative description.[51]

Subgroup analysis

When possible, the following subgroups will be made:

- Subgroups according to the imaging technique used with the aim of reduce possible heterogeneity arising from this methodological variety.
- Subgroups according to the treatment received.

For peer review only - http://bmjopen.bmj.com/site/about/guidelines.xhtml

- Subgroups according to the presence/absence of HT.
- Subgroups according to the mRS 90 days value: (1) mRS 0-1; (2) mRS 2-5

We will compare the permeability values among the subgroups and, if possible, correlate these with the different features/predictors of stroke.

Meta-bias(es)

To assess publication bias, we will conduct a funnel plot following the recommendation of the Cochrane Handbook for Systematic Reviews of Interventions[48] and a complemental Egger's test in order to quantify the funnel plot's asymmetry.

Confidence in cumulative evidence

The strength of the body evidence will be assessed using the Grading Recommendations Assessment, Development and Evaluation (GRADE).[48]

Patient and public involvement

This is a protocol for a systematic review that will be based on previously published data, therefore no participant recruitment will take place. The involvement of participants on the recruitment and dissemination of results is not applicable.

ETHICS AND DISSEMINATION

This work will be based in data that is public and already published, therefore an ethical approval would not be necessary. The result obtained from this work will be published in a peer-reviewed journal and disseminated in relevant conferences. If any amendments are needed due to deviations from this protocol in the execution of the study, these amendments will be recorded and noted in the publication.

Authors' contributions: JSF and SBC formulated the idea for this systematic review. SBC drafted the protocol guided by JSF and HD. JSF, HD and LF reviewed all manuscript versions.

Funding: This work is framed within the NANOSTEM project. This project has received funding from the European Union's Horizon 2020 research and innovation program under grant agreement number 764958.

Competing interests' statement: None declared.

For peer review only

REFERENCES

1 GBD 2016 Stroke Collaborators. Global, regional, and national burden of stroke, 1990–2016: a systematic analysis for the Global Burden of Disease Study 2016. *Lancet Neurol* 2019;18:439–58.

2 Rajsic S, Gothe H, Borba HH, *et al.* Economic burden of stroke: a systematic review on post-stroke care. *Eur J Heal Econ* 2019;20:107–34.

3 Hoffmann A, Zhu G, Wintermark M. Advanced neuroimaging in stroke patients: Prediction of tissue fate and hemorrhagic transformation. *Expert Rev Cardiovasc Ther* 2012;10:515–24.

4 Matta R, Gonzalez AL. Stroke Repair via Biomimicry of the Subventricular Zone. *Front Mater* 2018;5.

5 Wey HY, Desai VR, Duong TQ. A review of current imaging methods used in stroke research. *Neurol Res* 2013;35:1092–102.

6 Barber PA. Magnetic resonance imaging of ischemia viability thresholds and the neurovascular unit. *Sensors* 2013;13:6981–7003.

7 Kassner A, Merali Z. Assessment of Blood-Brain Barrier Disruption in Stroke. *Stroke* 2015;46:3310–5.

8 Avsenik J, Bisdas S, Popovic KS. Blood-brain barrier permeability imaging using perfusion computed tomography. *Radiol Oncol* 2015;49:107–14.

9 Jiang X, Andjelkovic A, Zhu L, *et al.* Blood-brain barrier dysfunction and recovery after ischemic stroke. *Prog Neurobiol* 2018;163–164:144–71.

10 Wong AD, Ye M, Levy AF, *et al.* The blood-brain barrier: An engineering perspective. *Front Neuroeng* 2013;6:1–22.

11 Villringer K, Sanz Cuesta B, Ostwaldt AC, *et al.* DCE-MRI blood–brain barrier assessment in acute ischemic stroke. *Neurology* 2017;88:433–40.

12 Jiang Q, Ewing JR, Chopp M. MRI of Blood-Brain Barrier Permeability in Cerebral Ischemia. *Transl Stroke Res* 2012;3:56–64.

13 Yang Y, Rosenberg GA. Blood-brain barrier breakdown in acute and chronic cerebrovascular disease. *Stroke* 2011;42:3323–8.

14 De Simoni MG, Milia P, Barba M, *et al.* The inflammatory response in cerebral ischemia: Focus on cytokines in stroke patients. *Clin Exp Hypertens* 2002;24:535–42.

15 Mehta SL, Manhas N, Raghubir R. Molecular targets in cerebral ischemia for developing novel therapeutics. *Brain Res Rev* 2007;54:34–66.

16 Bernhardt J, Hayward KS, Kwakkel G, *et al.* Agreed definitions and a shared vision for new standards in stroke recovery research: The Stroke Recovery and Rehabilitation Roundtable taskforce. *Int J Stroke* 2017;12:444–50.

17 Carmichael ST. The 3 Rs of Stroke Biology: Radial, Relayed, and Regenerative. *Neurotherapeutics* 2016;13:348–59.

18 Heiss WD. The ischemic penumbra: How does tissue injury evolve? *Ann N Y Acad Sci* 2012;1268:26–34.

For peer review only

19 Chopp M, Zhang ZG, Jiang Q. Neurogenesis, angiogenesis, and MRI indices of functional recovery from stroke. *Stroke* 2007;38(Sppl2):827–31.

20 Ding GL, Jiang Q, Li L, *et al.* Angiogenesis Detected After Embolic Stroke in Rat Brain Using Magnetic Resonance T2*WI. *Stroke* 2008;39:1563–8.

21 Sargento-Freitas J, Aday S, Nunes C, *et al.* Endothelial progenitor cells enhance blood-brain barrier permeability in subacute stroke. *Neurology* 2018;90:e127–34.

22 Hone EA, Hu H, Sprowls SA, *et al.* Biphasic Blood-Brain Barrier Openings after Stroke. *Neurol Disord Stroke Int* 2018;1:11–4.

23 Rosenberg GA, Estrada EY, Dencoff JE. Matrix metalloproteinases and TIMPs are associated with blood-brain barrier opening after reperfusion in rat brain. *Stroke* 1998;29:2189–95.

24 Huang ZG, Xue D, Preston E, *et al.* Biphasic opening of the blood-brain barrier following transient focal ischemia: Effects of hypothermia. *Can J Neurol Sci* 1999;26:298–304.

25 Liu P, Zhang R, Liu D, *et al.* Time-course investigation of blood–brain barrier permeability and tight junction protein changes in a rat model of permanent focal ischemia. *J Physiol Sci* 2018;68:121–7.

26 Pillai DR, Pharm M, Dittmar MS, *et al.* Cerebral ischemia-reperfusion injury in rats-A 3T MRI study on biphasic Blood-brain barrier opening and the dynamics od edema foramtion. *J Cereb Blood Flow Metab* 2009;29:1846–55.

27 Lin CY, Chang C, Cheung WM, *et al.* Dynamic changes in vascular permeability, cerebral blood volume, vascular density, and size after transient focal cerebral ischemia in rats: Evaluation with contrast-enhanced magnetic resonance imaging. *J Cereb Blood Flow Metab* 2008;28:1491–501.

28 Strbian D, Durukan A, Pitkonen M, *et al.* The blood-brain barrier is continuously open for several weeks following transient focal cerebral ischemia. *Neuroscience* 2008;153:175–81.

29 Durukan A, Marinkovic I, Strbian D, *et al.* Post-ischemic blood-brain barrier leakage in rats: One-week follow-up by MRI. *Brain Res* 2009;1280:158–65.

30 Abo-Ramadan U, Durukan A, Pitkonen M, *et al.* Post-ischemic leakiness of the blood-brain barrier: A quantitative and systematic assessment by Patlak plots. *Exp Neurol* 2009;219:328–33.

31 Merali Z, Huang K, Mikulis D, *et al.* Evolution of blood-brain-barrier permeability after acute ischemic stroke. *PLoS One* 2017;12:1–11.

32 Liu HS, Chung HW, Chou MC, *et al.* Effects of microvascular permeability changes on contrast-enhanced t1 and pharmacokinetic mr imagings after ischemia. *Stroke* 2013;44:1872–7.

33 Donahue MJ, Achten E, Cogswell PM, *et al.* Consensus statement on current and emerging methods for the diagnosis and evaluation of cerebrovascular disease. *J Cereb Blood Flow Metab* 2018;38:1391–417.

34 Mikulis DJ. Functional cerebrovascular imaging in brain ischemia: Permeability, reactivity, and functional MR imaging. *Neuroimaging Clin N Am* 2005;15:667–80.

35 Yen P, Cobb A, Shankar JJS. Does computed tomography permeability predict

For peer review only

hemorrhagic transformation after ischemic stroke? *World J Radiol* 2016;8:594.

36 Leigh R, Christensen S, Campbell BCV, *et al.* Pretreatment blood-brain barrier disruption and post-endovascular intracranial hemorrhage. *Neurology* 2016;87:263–9.

37 Nguyen GT, Coulthard A, Wong A, *et al.* Measurement of blood-brain barrier permeability in acute ischemic stroke using standard first-pass perfusion CT data. *NeuroImage Clin* 2013;2:658–62.

38 Chen H, Zhu G, Liu N, *et al.* Applications and development of permeability imaging in ischemic stroke (Review). *Exp Ther Med* 2018;16:2203–7.

39 Cuenod CA, Balvay D. Perfusion and vascular permeability: Basic concepts and measurement in DCE-CT and DCE-MRI. *Diagn Interv Imaging* 2013;94:1187–204.

40 Gordon Y, Partovi S, Müller-Eschner M, *et al.* Dynamic contrast-enhanced magnetic resonance imaging: fundamentals and application to the evaluation of the peripheral perfusion. *Cardiovasc Diagn Ther* 2014;4:147–64.

41 Harrigan M, Leonardo J, Gibbons K, *et al.* CT Perfusion Cerebral Blood Flow Imaging in Neurological Critical Care. *Neurocrit Care* 2005;2:352-66.

42 Kassner A, Mandell DM, Mikulis DJ. Measuring Permeability in Acute Ischemic Stroke. *Neuroimaging Clin N Am* 2011;21:315–25.

43 Jaillard A, Cronu C, Durieux A, *et al.* Hemorrhagic Transformation in Acute Ischemic Stroke The MAST-E Study Assia. *Stroke* 1999;30:1326–32.

44 Suh CH, Jung SC, Cho SJ, *et al.* MRI for prediction of hemorrhagic transformation in acute ischemic stroke: a systematic review and meta-analysis. *Acta radiol* 2019;18:284185119887593.

45 Suh CH, Jung SC, Cho SJ, *et al.* Perfusion CT for prediction of hemorrhagic transformation in acute ischemic stroke: a systematic review and meta-analysis. *Eur Radiol* 2019;29:4077–87.

46 Ryu WHA, Avery MB, Dharampal N, *et al.* Utility of perfusion imaging in acute stroke treatment: a systematic review and meta-analysis. *J Neurointerv Surg* 2017;9:1012–6.

47 Liberati A, Altman DG, Tetzlaff J, et al. The PRISMA statement for reporting systematic reviews and meta-analyses of studies that evaluate health care interventions: explanation and elaboration. *PLoS Med* 2009 21;6:e1000100.

48 Higgins J, Green S. Cochrane Handbook for Systematic Reviews of Interventions Version 5.1.0 [updated March 2011]. The Cochrane Collaboration 2011. Available at: www.cochrane-handbook.org.

49 Wells G, Shea B, O'Connell D, *et al.* The Newcastle-Ottawa Scale (NOS) for assessing the quality of nonrandomised studies in meta-analyses. 2008. Available at: http://www.ohri.ca/programs/clinical_epidemiology/oxford.asp. (Accessed: 5th August 2019)

50 Higgins J, Thompson S, Deeks J, *et al.* Measuring inconsistency in meta-analyses. *BMJ* 2003;327:557–60.

51 Thanvi BR, Treadwell S, Robinson T. Haemorrhagic transformation in acute ischaemic stroke following thrombolysis therapy: Classification, pathogenesis and risk factors. *Postgrad Med J* 2008;84:361–7.

For peer review only

Legends

Figure 1. Flow-chart diagram presenting the selection process for the studies.

For peer review only

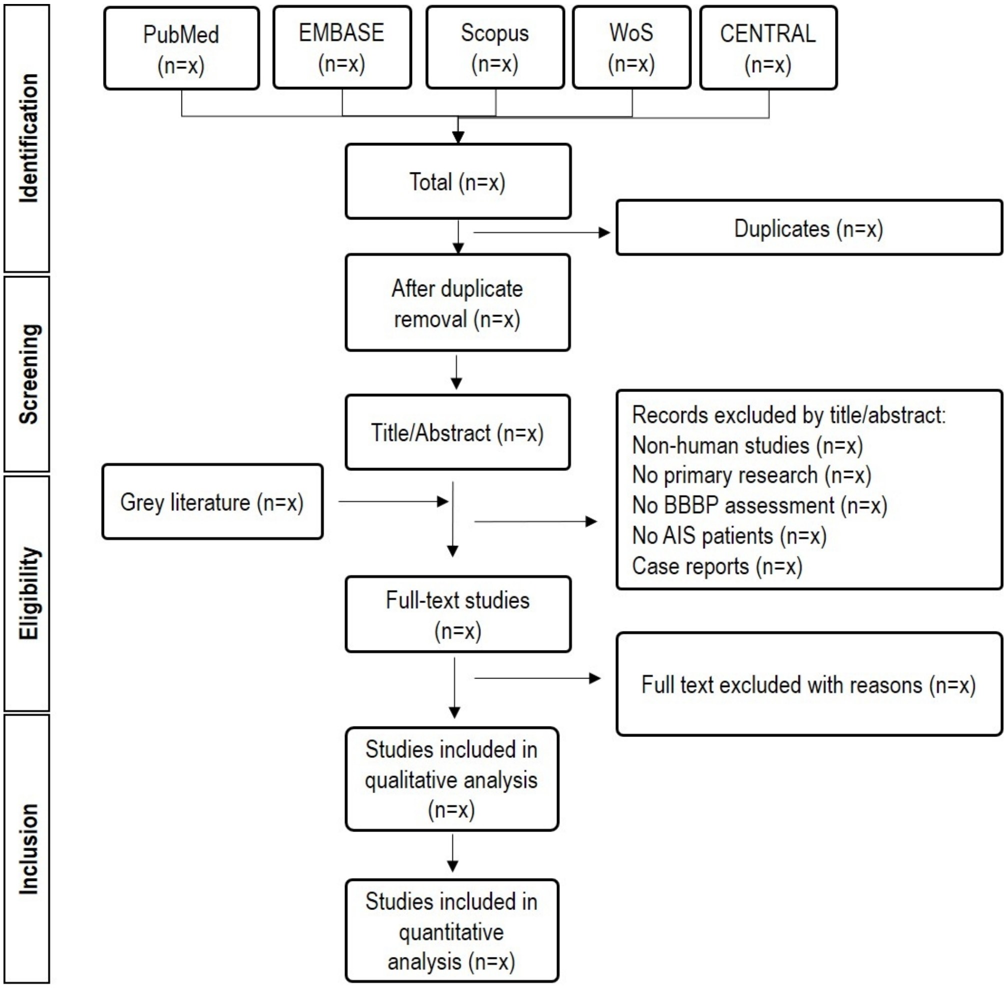

Figure 1. Flow-chart diagram presenting the selection process for the studies.

178x175mm (150 x 150 DPI)

PRISMA-P (Preferred Reporting Items for Systematic review and Meta-Analysis Protocols) 2015 checklist: recommended items to address in a systematic review protocol*

Section and topic	Item No	Checklist item
**ADMINISTRATIVE INFORMATION**		
Title: **pg.1**		
Identification **pg.1**	1a	Identify the report as a protocol of a systematic review
Update **n/a**	1b	If the protocol is for an update of a previous systematic review, identify as such
Registration **pg.2**	2	If registered, provide the name of the registry (such as PROSPERO) and registration number
Authors: **pg.1**		
Contact **pg.1**	3a	Provide name, institutional affiliation, e-mail address of all protocol authors; provide physical mailing address of corresponding author
Contributions **pg.9**	3b	Describe contributions of protocol authors and identify the guarantor of the review
Amendments **pg.9**	4	If the protocol represents an amendment of a previously completed or published protocol, identify as such and list changes; otherwise, state plan for documenting important protocol amendments
Support: **pg.9**		
Sources	5a	Indicate sources of financial or other support for the review
Sponsor	5b	Provide name for the review funder and/or sponsor
Role of sponsor or funder	5c	Describe roles of funder(s), sponsor(s), and/or institution(s), if any, in developing the protocol
**INTRODUCTION**		
Rationale **pg.3**	6	Describe the rationale for the review in the context of what is already known
Objectives **pg.4**	7	Provide an explicit statement of the question(s) the review will address with reference to participants, interventions, comparators, and outcomes (PICO)
**METHODS**		
Eligibility criteria **pg.4**	8	Specify the study characteristics (such as PICO, study design, setting, time frame) and report characteristics (such as years considered, language, publication status) to be used as criteria for eligibility for the review
Information sources **pg.5**	9	Describe all intended information sources (such as electronic databases, contact with study authors, trial registers or other grey literature sources) with planned dates of coverage
Search strategy **pgs.5-6**	10	Present draft of search strategy to be used for at least one electronic database, including planned limits, such that it could be repeated
Study records:		
Data management **pg.6**	11a	Describe the mechanism(s) that will be used to manage records and data throughout the review

For peer review only

Selection process **pg.6**	11b	State the process that will be used for selecting studies (such as two independent reviewers) through each phase of the review (that is, screening, eligibility and inclusion in meta-analysis)
Data collection process **pg.7**	11c	Describe planned method of extracting data from reports (such as piloting forms, done independently, in duplicate), any processes for obtaining and confirming data from investigators
Data items **pg.7**	12	List and define all variables for which data will be sought (such as PICO items, funding sources), any pre-planned data assumptions and simplifications
Outcomes and prioritization **pg.7**	13	List and define all outcomes for which data will be sought, including prioritization of main and additional outcomes, with rationale
Risk of bias in individual studies **pg.8**	14	Describe anticipated methods for assessing risk of bias of individual studies, including whether this will be done at the outcome or study level, or both; state how this information will be used in data synthesis
Data synthesis **pg.8**	15a	Describe criteria under which study data will be quantitatively synthesised
	15b	If data are appropriate for quantitative synthesis, describe planned summary measures, methods of handling data and methods of combining data from studies, including any planned exploration of consistency (such as I^2, Kendall's τ)
	15c	Describe any proposed additional analyses (such as sensitivity or subgroup analyses, meta-regression)
	15d	If quantitative synthesis is not appropriate, describe the type of summary planned
Meta-bias(es) **pg. 9**	16	Specify any planned assessment of meta-bias(es) (such as publication bias across studies, selective reporting within studies)
Confidence in cumulative evidence **pg.9**	17	Describe how the strength of the body of evidence will be assessed (such as GRADE)

*** It is strongly recommended that this checklist be read in conjunction with the PRISMA-P Explanation and Elaboration (cite when available) for important clarification on the items. Amendments to a review protocol should be tracked and dated. The copyright for PRISMA-P (including checklist) is held by the PRISMA-P Group and is distributed under a Creative Commons Attribution Licence 4.0.**

From: Shamseer L, Moher D, Clarke M, Ghersi D, Liberati A, Petticrew M, Shekelle P, Stewart L, PRISMA-P Group. Preferred reporting items for systematic review and meta-analysis protocols (PRISMA-P) 2015: elaboration and explanation. BMJ. 2015 Jan 2;349(jan02 1):g7647.

For peer review only

BMJ Open

Permeability of the blood brain barrier through the phases of ischemic stroke and relation with clinical outcome: protocol for a systematic review

Journal:	*BMJ Open*
Manuscript ID	bmjopen-2020-039280.R2
Article Type:	Protocol
Date Submitted by the Author:	15-Jul-2020
Complete List of Authors:	Bernardo-Castro, Sara; Centro Hospitalar e Universitário de Coimbra EPE, Stroke Unit<br>Donato, Helena; Centro Hospitalar e Universitario de Coimbra EPE, Documentation Service<br>Ferreira, Lino; Center for Neurosciences and Cell Biology, Universidade de Coimbra; Universidade de Coimbra, Faculdade de Medicina<br>Sargento-Freitas, João; Centro Hospitalar e Universitario de Coimbra EPE, Stroke Unit; Universidade de Coimbra, Faculdade de Medicina
**Primary Subject Heading**:	Neurology
Secondary Subject Heading:	Radiology and imaging
Keywords:	Stroke < NEUROLOGY, Magnetic resonance imaging < RADIOLOGY & IMAGING, Neuroradiology < RADIOLOGY & IMAGING, VASCULAR MEDICINE, Computed tomography < RADIOLOGY & IMAGING

SCHOLARONE™
Manuscripts

I, the Submitting Author has the right to grant and does grant on behalf of all authors of the Work (as defined in the below author licence), an exclusive licence and/or a non-exclusive licence for contributions from authors who are: i) UK Crown employees; ii) where BMJ has agreed a CC-BY licence shall apply, and/or iii) in accordance with the terms applicable for US Federal Government officers or employees acting as part of their official duties; on a worldwide, perpetual, irrevocable, royalty-free basis to BMJ Publishing Group Ltd ("BMJ") its licensees and where the relevant Journal is co-owned by BMJ to the co-owners of the Journal, to publish the Work in this journal and any other BMJ products and to exploit all rights, as set out in our licence.

The Submitting Author accepts and understands that any supply made under these terms is made by BMJ to the Submitting Author unless you are acting as an employee on behalf of your employer or a postgraduate student of an affiliated institution which is paying any applicable article publishing charge ("APC") for Open Access articles. Where the Submitting Author wishes to make the Work available on an Open Access basis (and intends to pay the relevant APC), the terms of reuse of such Open Access shall be governed by a Creative Commons licence – details of these licences and which Creative Commons licence will apply to this Work are set out in our licence referred to above.

Other than as permitted in any relevant BMJ Author's Self Archiving Policies, I confirm this Work has not been accepted for publication elsewhere, is not being considered for publication elsewhere and does not duplicate material already published. I confirm all authors consent to publication of this Work and authorise the granting of this licence.

For peer review only

Title: Permeability of the blood brain barrier through the phases of ischemic stroke and relation with clinical outcome: protocol for a systematic review

Authors: Sara Bernardo-Castro[1], Helena Donato[2], Lino Ferreira[3,4], João Sargento-Freitas[1,4]

Affiliations:

1. **Stroke Unit, Centro Hospitalar e Universitário de Coimbra, Coimbra, Portugal**
2. **Documentation Department, Centro Hospitalar e Universitário de Coimbra, Coimbra, Portugal**
3. **Center for Neuroscience and Cell Biology, Universidade de Coimbra, Coimbra, Portugal**
4. **Faculdade de Medicina da Universidade de Coimbra, Coimbra, Portugal**

Corresponding author:

João Sargento-Freitas

Stroke Unit, Centro Hospitalar e Universitário de Coimbra, Coimbra, Portugal

Mail: jsargentof@hotmail.com

Word count: 2725

For peer review only

ABSTRACT

Introduction: Ischemic stroke is the most prevalent type of stroke and is characterized by a myriad of pathological events triggered by a vascular arterial occlusion. Disruption of the blood-brain barrier (BBB) is a key pathological event that may lead to fatal outcomes. However, it seems to follow a multiphasic pattern that has been associated with distinct biological substrates and possibly contrasting outcomes. Addressing the BBB permeability along the different phases of stroke through imaging techniques could lead to a better understanding of the disease, improved patient selection for specific treatments and development of new therapeutic modalities and delivery methods. This systematic review will aim to comprehensively summarize the existing evidence regarding the evolution of the BBB permeability values during the different phases of an acute ischemic stroke and correlate this event with the clinical outcome of the patient.

Methods and analysis: We will conduct a computerized search on Medline, EMBASE, CENTRAL, Scopus and Web of Science. In addition, grey literature and ClinicalTrials.gov will be scanned. We will include randomized control trials, cohort, cross-sectional and case-controlled studies on humans that quantitatively assess the BBB permeability in stroke. Retrieved studies will be independently reviewed by two authors and any discrepancies will be resolved by consensus or with a third reviewer. Reviewers will extract the data and assess the risk of bias of the selected studies. If possible, data will be combined in a quantitative meta-analysis following the guidelines provided by Cochrane Handbook for Systematic Reviews of Interventions. We will assess cumulative evidence using the Grading of Recommendations, Assessment, Development and Evaluation (GRADE) approach.

Ethics and dissemination: Ethical approval is not needed. All data used for this work is publicly available. The result obtained from this work will be published in a peer-reviewed journal and disseminated in relevant conferences.

PROSPERO registration: CRD42019147314

Strengths and limitations of this study

- To our knowledge this is the first systematic review that will focus on the progression of the BBB permeability during AIS and its clinical consequences.
- This protocol has been developed following Preferred Reporting Items for Systematic Review for systematic review protocols (PRISMA-P).
- This systematic review could help to guide conventional recanalization treatments outside the therapeutic windows and other innovative delivery treatments in later stage of AIS.
- We will include all types of studies, not imposing any restriction on language or year.
- The vast heterogeneity that can arise from the use of different BBBP imaging techniques, the pharmacokinetic models used and the different permeability parameters yield from each technique, may prevent a quantitative meta-analysis from being conducted.

For peer review only

INTRODUCTION

Stroke is the second leading cause of mortality and morbidity worldwide.[1–4] Every year around 14 million people suffer a stroke, 5.5 million of which die[1] and another 5 million stay permanently disabled, representing a significant concern for public health and society.[2] Acute ischemic strokes (AIS) account for approximately 85% of all strokes,[1,3,4] they restrict blood flow to a specific region of the brain leading to death of the compromised tissue.[5,6] Currently, the only available and effective treatment to limit this situation is recanalization therapy, to restore the normal blood flow,[7] but these therapies can only be given to less than 5% of patients due to their narrow therapeutic window.[4,7] Treating patients outside this window, could contribute to additional tissue damage and increase in the risk of hemorrhagic transformation (HT).[7,8]

The blood-brain barrier (BBB) is a dynamic physiological structure that constitutes an interface between the vasculature system and the neural tissues. It regulates the transport of substances in a bi-directional way[9] and protects the central nervous system (CNS) from unwanted compounds, playing a crucial role in maintaining its homeostasis.[9,10] During the process of ischemia the BBB undergoes a dysfunction[9,11] that leads to an increase of its permeability,[10] enabling the passage of large molecules, fluids and blood into the brain interstitium.[8,9] This pathological leakage is associated with a worst outcome after AIS[3,8,12] and is known to persists for several days[7] following a time-course mediated by complex pathophysiological events with different clinical implications.[13–15] During the first hours after stroke, namely the hyperacute and acute phases, the insult triggers ischemic cell death which leads to a higher risk of HT.[16,17] In a late acute/early subacute stage, the BBB is disrupted due to the secondary ischemic injury which causes inflammatory cell infiltration and tissue scaring and a further BBB permeability (BBBP) increase.[17,18] In a later subacute stage, the BBBP relies on physiological recovery processes such as neoangiogenesis, as demonstrated in both animals[19,20] and humans.[21] Whereas the existence of this permeability process is unequivocal, its concrete evolution is not yet certain.

Several longitudinal animal studies have tried to explain this event. Some studies point to an “open-close-open” biphasic pattern in which the BBB has increased permeability at a first stage followed by a return to normal values and a second permeability increase.[22–24] Nonetheless these studies show differences on the open/close times and more recent literature points to a more continuous opening of the BBB with biphasic permeability peaks but without total closing [25–28]. A first BBBP increase has been shown to appear as soon as 3/6 hours after occlusion, followed by a decrease but not a total recovery, and a second peak at the early subacute stage [26,27,29]. Studies extending the BBBP quantification time-points, have reported a further increased permeability up to one[27,29], three[27] and even five weeks[28,30] after occlusion, suggesting that the BBB could remain open until months after the onset of stroke.

Very few human studies have focused on studying this evolution[11,21,31,32] and even though these studies point to a continuous opening of the BBB, they do not offer a clear and collective evidence on the magnitude of this opening in the different phases. A quantitative assessment of the BBB disruption through its permeability could add valuable information in the evaluation of AIS patients.

Three main imaging techniques are used to evaluate BBBP: computed tomography (CT), magnetic resonance imaging (MRI) and positron emission tomography (PET).[33] Nonetheless,

For peer review only

due to the limited availability of PET, the "permeability imaging" term is used mainly for MRI and CT.[7] These specific imaging tools are able to measure the BBBP in vivo in a non-invasive manner.[3,9,11,34–37] In short, these imaging modalities quantify the rate and amount at which a specific contrast agent leaves the blood stream and enters the brain parenchyma[12,34,38] using mathematical models able to describe the physiological characteristics of the BBB such as vessel permeability, vessel surface area product and tissue volume fraction.[11,39,40] In clinical practice this information has been used as a diagnostic tool for differential diagnosis, and also to support decisions for safer and improved recanalization therapies for stroke patients in an extended time window.[7–9,11,38,41–43]

Nonetheless, although there are important systematic reviews that focus on the implications of imaging and increased permeability on stroke outcome,[44,45] and on the utility of perfusion imaging in determining treatment eligibility in patients with acute stroke,[46] to date there are no systematic reviews, to our knowledge, focusing on the development of the BBBP during the phases of AIS.

Ideally, this knowledge would help not only in extending the treatment window, but also in the development of future treatment options such as delivery system strategies for neuroprotective or neurorestorative treatments that aim to use the BBB as a therapeutic vehicle or target. Therefore, there is a need to perform a systematic review and meta-analysis on the BBBP dynamics after AIS to gather larger sample sizes of patients and create a concrete understanding of the subject. This systematic review will provide an insight on the evolution of the permeability of the blood brain barrier in patients affected by AIS through the different stages of the stroke and its relevance in the patient outcome and treatment.

Objective

The main objective of this work is to carry out a systematic review and meta-analysis on the blood-brain barrier permeability during the different phases of an acute ischemic stroke with the aim of assessing its evolution through time and its correlation with clinical outcome.

METHODS

Eligibility criteria

This work will identify randomized controlled trials, cohort studies (prospective or retrospective), cross-sectional studies and case-controlled studies that quantify BBBP in patients suffering from AIS. Studies fulfilling the eligibility criteria shown in Table 1 will be selected for further review. If more than one article reports the same study, the article with the largest sample size or reporting more relevant data for our specific aim will be selected. No restriction regarding publication year will be set; therefore, we will be including studies since inception to 31st July 2019. In addition, no language restriction will be applied. If a study in a non-understandable language is obtained, we will consider its suitability for our study by its English abstract and if the information is interesting enough to be included, the paper will be sent to a professional translator.

For peer review only

Table 1. Inclusion/exclusion criteria for study selection

**Inclusion criteria**	**Exclusion criteria**
Studies on living humans	Non-human studies
Acute ischemic stroke	Lacunar strokes (subcortical ischemic lesion with a diameter under 15mm in CT or 20mm in MRI)
BBBP evaluation through neuroimaging	Mild stroke (NIHSS below 6)
Studies with a follow up for clinical outcome	Hemorrhagic stroke
	BBBP evaluation through non-imaging techniques
	BBBP evaluation in other non-AIS diseases
	No primary research
	Reports just defining a study protocol
	Case-report studies
	Studies not reporting time from onset to imaging
	Studies no reporting contralateral permeability values

Information sources

We will conduct a comprehensive computerized literature search strategy to find the studies that will take part in this systematic review. We will search for both published and unpublished studies in the following data bases: PubMed/MEDLINE, EMBASE, Web of Science, Scopus and Cochrane Central Register of Controlled Trials (CENTRAL). Other electronic platforms such as ClinicalTrials.gov will be scanned to keep-up with ongoing or unpublished clinical trials. If any relevant unpublished trial is found, the corresponding author listed will be contacted to obtain the required information. If no response is given or, if the author decides not to share the data, this will be listed as the reason for exclusion of said trial. In addition to this electronic search, a supplementary search of the grey literature will be conducted with the aim of include all possible existing articles on the subject. No pre-prints will be included on the study.

Search strategy

The search strategy will include the following terms and all of its variants in multiple combinations adapted to each one of the data bases regarding its own special requirements as shown in Table 2: 'stroke', 'permeability', 'blood brain barrier', 'imaging', 'neuroimaging'. The search of the grey literature will include a by-hand search of relevant articles in the listed bibliography of the selected studies and important reviews on the subject, conference papers and a google search of the used terms.

Table 2. Retrieval search strategy

PubMed	
**Query**	**Search**
#1	"Stroke"[MeSH Terms] OR "stroke" OR "cerebral stroke" OR "ischemic stroke" OR "acute stroke" OR "acute ischemic stroke" OR "apoplexy" OR "cerebral apoplexy" OR "cerebrovascular accident" OR "acute cerebrovascular accident" OR "brain vascular accident" OR "CVA"
#2	"Blood-Brain Barrier"[MeSH Terms] OR "Blood-Brain Barrier" OR "Blood Brain Barrier" OR "Brain-Blood Barrier" OR "Hemato Encephalic Barrier" OR "Hemato-Encephalic Barrier"
#3	"Permeability"[MeSH Terms] OR "Permeability" OR "Leakage"
#4	"Diagnostic Imaging"[Mesh] OR "Neuroimaging" [MeSH Terms] OR "Neuroimaging" OR "Brain Imaging" OR "Magnetic Resonance Imaging"[MeSH Terms] OR "Magnetic Resonance Imaging" OR "MRI" OR "MRI scan" OR "Functional MRI" OR "fMRI" OR "Computed Tomography Angiography"[MeSH Terms] OR "Computed Tomography Angiography" OR "Computed Tomography" OR "CT" OR "CT angiography" OR " dynamic contrast enhanced MRI" OR "dynamic susceptibility contrast MRI" OR "computed tomography perfusion"

For peer review only

#5	Search #1 AND #2 AND #3 AND #4
EMBASE	
#1	'cerebrovascular accident'/exp OR 'brain ischemia'/exp OR 'cerebrovascular accident' OR 'stroke patient' OR 'brain ischemia' OR 'stroke' OR 'acute ischemic stroke' OR 'ischemic stroke' OR 'apoplexy' OR 'cerebral apoplexy' OR 'brain apoplexy'
#2	'blood brain barrier'/exp OR 'blood brain barrier' OR 'hemato encephalic barrier' OR 'hemato-encephalic barrier'
#3	'permeability'/exp OR ‘permeability’ OR ‘leakage’
#4	'diagnostic imaging'/exp OR 'neuroimaging'/exp OR 'functional magnetic resonance imaging'/exp OR 'nuclear magnetic resonance imaging'/exp OR 'nuclear magnetic resonance'/exp OR 'computer assisted tomography'/exp OR neuroimaging' OR 'nuclear magnetic resonance imaging' OR 'mri' OR 'functional magnetic imaging' OR 'fmri' OR 'computer assisted tomography' OR 'computed tomographic angiography' OR 'ct' OR ‘dynamic contrast enhanced MRI’ OR ‘dynamic susceptibility contrast MRI’ OR ‘computed tomography perfusion’
#5	#1 AND #2 AND #3 AND #4
Scopus	
#1	TITLE-ABS-KEY ("stroke") OR TITLE-ABS-KEY ("ischemic stroke") OR TITLE-ABS-KEY ("acute ischemic stroke") OR TITLE-ABS-KEY ("cerebral apoplexy") OR TITLE-ABS-KEY ("cerebrovascular accident") OR TITLE-ABS-KEY ("acute cerebrovascular accident") OR TITLE-ABS-KEY ("brain apoplexy") OR TITLE-ABS-KEY ("CVA")
#2	("blood-brain barrier") OR TITLE-ABS-KEY ("hemato encephalic barrier") OR TITLE-ABS-KEY ("blood brain barrier") OR TITLE-ABS-KEY ("hemato-encephalic barrier")
#3	TITLE-ABS-KEY (“permeability”) OR TITLE-ABS-KEY (“leakage”)
#4	TITLE-ABS-KEY ("neuroimaging") OR TITLE-ABS-KEY ("magnetic resonance imaging") OR TITLE-ABS-KEY ("functional magnetic resonance imaging") OR TITLE-ABS-KEY ("MRI") OR TITLE-ABS-KEY ("fMRI") OR TITLE-ABS-KEY ("computed tomography") OR TITLE-ABS-KEY ("computed tomography angiography") OR TITLE-ABS-KEY ("CT") OR TITLE-ABS-KEY (“dynamic contrast enhanced MRI") OR TITLE-ABS-KEY ("dynamic susceptibility contrast MRI") OR TITLE-ABS-KEY ("computed tomography perfusion"))
CENTRAL	
#1	('blood brain barrier' OR 'hemato encephalic barrier' OR 'hemato-encephalic barrier') AND ('permeability' OR ‘leakage’) AND ('cerebrovascular accident' OR 'stroke patient' OR 'brain ischemia' OR 'stroke' OR 'acute ischemic stroke' OR 'ischemic stroke' OR 'apoplexy' OR 'cerebral apoplexy' OR 'brain apoplexy') AND ('neuroimaging' OR 'nuclear magnetic resonance imaging' OR 'mri' OR 'functional magnetic imaging' OR 'fmri' OR 'computer assisted tomography' OR 'computed tomographic angiography' OR 'ct' OR ‘dynamic contrast enhanced MRI’ OR ‘dynamic susceptibility contrast MRI’ OR ‘computed tomography perfusion’)
Web of Science	
#1	TS=('blood brain barrier' OR 'hemato encephalic barrier' OR 'hemato-encephalic barrier') AND TS=('permeability' OR ‘leakage’) AND TS=('cerebrovascular accident' OR 'stroke patient' OR 'brain ischemia' OR 'stroke' OR 'acute ischemic stroke' OR 'ischemic stroke' OR 'apoplexy' OR 'cerebral apoplexy' OR 'brain apoplexy') AND TS=('neuroimaging' OR 'nuclear magnetic resonance imaging' OR 'mri' OR 'functional magnetic imaging' OR 'fmri' OR 'computer assisted tomography' OR 'computed tomographic angiography' OR 'ct' OR ‘dynamic contrast enhanced MRI’ OR ‘dynamic susceptibility contrast MRI’ OR ‘computed tomography perfusion’)

Data management

All publications arising from the literature search conducted, will be imported to the Mendeley citation software where duplicates will be managed and erased and titles/abstracts of all records will be scanned.

Selection process

Two independent reviewers will conduct the selection process. All records identified in the search stage will be screened by title/abstract and studies clearly not matching the criteria will be discarded. The remaining studies will be full-text reviewed and included or discarded according to the inclusion/exclusion criteria. Any disagreement between the reviewers will be resolved by consensus or by a third one if necessary. Reasons for the exclusion of full text records

For peer review only

will be recorded. Details on the selection process of the studies will be documented into a flow chart following the Preferred Reporting Items for Systematic Reviews and Meta-Analyses (PRIMSA)[47] as presented in Figure 1.

Data collection process

To ensure that all relevant information is captured, and to minimize the risk of bias, two reviewers will independently extract the information from the studies following the same pilot form. Any disagreement will be resolved by consensus. The data extracted will be reviewed and validated by a third reviewer.

Data items

Four main categories of data will be extracted from all studies selected: (1) Features of the study; (2) Patients characteristics; (3) Intervention; (4) Outcome. Among these categories a number of items will be collected as presented in Table 3.

Table 3. Data items to be collected from the selected studies

Features of the study	Patient characteristics	Intervention	Outcome
Title, author	Age, gender	Time form onset to imaging	Permeability values
Study design	Co-morbidities	Imaging characteristics	Final lesion (volume)
Recruitment procedure and duration	NHISS at admission	BBBP assessment characteristics	Follow up (length, number)
Number of participants	Stroke etiology (TOAST classification)	Reperfusion treatment given	Clinical outcome (NHISS and mRS)
Imaging modality	Vascular territory		Hemorrhagic transformation

Since the main aim of this work is to study the BBBP values in the different phases of stroke, we will form the following groups according to time from onset to imaging reported in each study:

1. Hyperacute stage: 6 hours or less.
2. Acute stage: between 6-48 hours.
3. Subacute stage: between 3-9 days.
4. Chronic stage: 30 or more days.

For any study reporting more than one BBBP measurement, each of the measurements will be considered as an independent study and will be placed in the corresponding group according to the time-points established above. These values will be identified as author, year followed by the name of the corresponding stage.

Outcomes and prioritization

This work has three primary outcomes: (1) the comparison of the quantitative permeability values across time after stroke; (2) The association between the different blood brain barrier permeability values and the functional outcome of acute stroke patients; (3) Association between permeability values and the recanalization treatment given.

When and if possible, the following secondary outcomes will be measured: (1) The association between the different blood brain barrier permeability values and hemorrhagic transformation;

For peer review only - http://bmjopen.bmj.com/site/about/guidelines.xhtml

(2) Association between any clinical feature/stroke predictor (age, hypertension, diabetes etc.) and the BBB permeability.

Risk of bias in individual studies

With the aim of minimizing bias, the methodological quality of all studies included in the systematic review, will be assessed independently by two reviewers. Since we will be including diverse types of studies, we will use different tools to assess the risk of bias depending of the characteristics of the studies, tuning these tools if necessary. For the RCT we will be using the Cochrane Collaboration's tool for assessing risk of bias in randomized trials.[48] This tool covers seven sources of bias: (1) random sequence generation; (2) allocation concealment; (3) blinding of participants and personnel; (4) blinding of outcome assessment; (5) incomplete outcome data; (6) selective reporting and (7) other bias. The risk of bias for each domain will be graded as high, low or unclear based on the relevant information extracted from each study. Low risk of bias will be given to the study if all of the domains are marked as low risk; intermediate risk of bias will be given when at least one of the domains is graded with unclear risk; high risk of bias will be given if high risk is given to at least one of the domains of the check list.

For non-randomize trials we will use the Newcastle-Ottawa Scale (NOS) for assessing the quality of non-randomized studies in meta-analysis.[49] These studies will be assessed based on three perspectives: (1) selection of study groups, (2) Comparability of the groups; (3) Ascertainment of exposure (in case-control studies) or outcome of interest (in cohort studies). This scale proposes a system in which a high-quality choice will be granted by a star. A maximum of 9 stars for study can be given. We will consider a score of 7 or more as low risk of bias/ high-quality and less than 5 will be considered as high risk of bias/poor quality.[50,51]

Any disagreement between the two reviewers will be resolved by consensus or by a third reviewer if necessary.

Data synthesis

This systematic review will include a quantitative meta-analysis if possible. The statistical analysis will be carried out taking into account the guidelines provided by Cochrane Handbook for Systematic Reviews of Interventions.[48] As our main outcome will be presented as continuous data (permeability values), we will use the mean difference (MD) or the standardized mean difference (SMD) and the respective 95% CI to combine the results. We will test the consistency and heterogeneity of the studies with the Higgins I^2 statistic that can also be used to describe heterogeneity among subgroups.[52] Following the direction given by Higgings et al[52] we will consider ≤25% as low heterogeneity, between 25 and 50% as moderate heterogeneity and >75% as high heterogeneity. If the I^2 value is ≤50% (low to moderate heterogeneity) we will use the fixed effect model for data synthesis, if it is greater than 50%, we will use the random effects model. If the heterogeneity values are over 75%, we will search for the possible sources of this high heterogeneity, including reviewing the methodological processes of the selected studies, and search for outliers or influential cases that may distort the results of the analysis. Any possible outlier or influential case, as well as studies presenting with poor methodological quality and/or a high or critical risk of bias, will be excluded in a further sensitivity analysis.

For peer review only

If we are not able to collect the appropriate outcome information or not enough studies are retrieved for the different stages, we will consider that a quantitative meta-analysis is not feasible and we will conduct a narrative description.[53]

Subgroup analysis

When possible, the following subgroups will be made:

- Subgroups according to the imaging technique used with the aim of reduce possible heterogeneity arising from this methodological variety.
- Subgroups according to the treatment received.
- Subgroups according to the presence/absence of HT.
- Subgroups according to the mRS 90 days value: (1) mRS 0-1; (2) mRS 2-5

We will compare the permeability values among the subgroups and, if possible, correlate these with the different features/predictors of stroke.

Meta-bias(es)

To assess publication bias, we will conduct a funnel plot following the recommendation of the Cochrane Handbook for Systematic Reviews of Interventions[48] and a complemental Egger's test in order to quantify the funnel plot's asymmetry.

Confidence in cumulative evidence

The strength of the body evidence will be assessed using the Grading Recommendations Assessment, Development and Evaluation (GRADE).[48]

Patient and public involvement

This is a protocol for a systematic review that will be based on previously published data, therefore no participant recruitment will take place. The involvement of participants on the recruitment and dissemination of results is not applicable.

ETHICS AND DISSEMINATION

This work will be based in data that is public and already published, therefore an ethical approval would not be necessary. The result obtained from this work will be published in a peer-reviewed journal and disseminated in relevant conferences. If any amendments are needed due to deviations from this protocol in the execution of the study, these amendments will be recorded and noted in the publication.

Authors' contributions: JSF and SBC formulated the idea for this systematic review. SBC drafted the protocol guided by JSF and HD. JSF, HD and LF reviewed all manuscript versions.

Funding: This work is framed within the NANOSTEM project. This project has received funding from the European Union's Horizon 2020 research and innovation program under grant agreement number 764958.

Competing interests' statement: None declared.

For peer review only

REFERENCES

1 GBD 2016 Stroke Collaborators. Global, regional, and national burden of stroke, 1990–2016: a systematic analysis for the Global Burden of Disease Study 2016. *Lancet Neurol* 2019;18:439–58.

2 Rajsic S, Gothe H, Borba HH, *et al.* Economic burden of stroke: a systematic review on post-stroke care. *Eur J Heal Econ* 2019;20:107–34.

3 Hoffmann A, Zhu G, Wintermark M. Advanced neuroimaging in stroke patients: Prediction of tissue fate and hemorrhagic transformation. *Expert Rev Cardiovasc Ther* 2012;10:515–24.

4 Matta R, Gonzalez AL. Stroke Repair via Biomimicry of the Subventricular Zone. *Front Mater* 2018;5.

5 Wey HY, Desai VR, Duong TQ. A review of current imaging methods used in stroke research. *Neurol Res* 2013;35:1092–102.

6 Barber PA. Magnetic resonance imaging of ischemia viability thresholds and the neurovascular unit. *Sensors* 2013;13:6981–7003.

7 Kassner A, Merali Z. Assessment of Blood-Brain Barrier Disruption in Stroke. *Stroke* 2015;46:3310–5.

8 Avsenik J, Bisdas S, Popovic KS. Blood-brain barrier permeability imaging using perfusion computed tomography. *Radiol Oncol* 2015;49:107–14.

9 Jiang X, Andjelkovic A, Zhu L, *et al.* Blood-brain barrier dysfunction and recovery after ischemic stroke. *Prog Neurobiol* 2018;163–164:144–71.

10 Wong AD, Ye M, Levy AF, *et al.* The blood-brain barrier: An engineering perspective. *Front Neuroeng* 2013;6:1–22.

11 Villringer K, Sanz Cuesta B, Ostwaldt AC, *et al.* DCE-MRI blood–brain barrier assessment in acute ischemic stroke. *Neurology* 2017;88:433–40.

12 Jiang Q, Ewing JR, Chopp M. MRI of Blood-Brain Barrier Permeability in Cerebral Ischemia. *Transl Stroke Res* 2012;3:56–64.

13 Yang Y, Rosenberg GA. Blood-brain barrier breakdown in acute and chronic cerebrovascular disease. *Stroke* 2011;42:3323–8.

14 De Simoni MG, Milia P, Barba M, *et al.* The inflammatory response in cerebral ischemia: Focus on cytokines in stroke patients. *Clin Exp Hypertens* 2002;24:535–42.

15 Mehta SL, Manhas N, Raghubir R. Molecular targets in cerebral ischemia for developing novel therapeutics. *Brain Res Rev* 2007;54:34–66.

16 Bernhardt J, Hayward KS, Kwakkel G, *et al.* Agreed definitions and a shared vision for new standards in stroke recovery research: The Stroke Recovery and Rehabilitation Roundtable taskforce. *Int J Stroke* 2017;12:444–50.

17 Carmichael ST. The 3 Rs of Stroke Biology: Radial, Relayed, and Regenerative. *Neurotherapeutics* 2016;13:348–59.

18 Heiss WD. The ischemic penumbra: How does tissue injury evolve? *Ann N Y Acad Sci* 2012;1268:26–34.

For peer review only

19 Chopp M, Zhang ZG, Jiang Q. Neurogenesis, angiogenesis, and MRI indices of functional recovery from stroke. *Stroke* 2007;38(Sppl2):827–31.

20 Ding GL, Jiang Q, Li L, *et al.* Angiogenesis Detected After Embolic Stroke in Rat Brain Using Magnetic Resonance T2*WI. *Stroke* 2008;39:1563–8.

21 Sargento-Freitas J, Aday S, Nunes C, *et al.* Endothelial progenitor cells enhance blood-brain barrier permeability in subacute stroke. *Neurology* 2018;90:e127–34.

22 Hone EA, Hu H, Sprowls SA, *et al.* Biphasic Blood-Brain Barrier Openings after Stroke. *Neurol Disord Stroke Int* 2018;1:11–4.

23 Rosenberg GA, Estrada EY, Dencoff JE. Matrix metalloproteinases and TIMPs are associated with blood-brain barrier opening after reperfusion in rat brain. *Stroke* 1998;29:2189–95.

24 Huang ZG, Xue D, Preston E, *et al.* Biphasic opening of the blood-brain barrier following transient focal ischemia: Effects of hypothermia. *Can J Neurol Sci* 1999;26:298–304.

25 Liu P, Zhang R, Liu D, *et al.* Time-course investigation of blood–brain barrier permeability and tight junction protein changes in a rat model of permanent focal ischemia. *J Physiol Sci* 2018;68:121–7.

26 Pillai DR, Pharm M, Dittmar MS, *et al.* Cerebral ischemia-reperfusion injury in rats-A 3T MRI study on biphasic Blood-brain barrier opening and the dynamics od edema foramtion. *J Cereb Blood Flow Metab* 2009;29:1846–55.

27 Lin CY, Chang C, Cheung WM, *et al.* Dynamic changes in vascular permeability, cerebral blood volume, vascular density, and size after transient focal cerebral ischemia in rats: Evaluation with contrast-enhanced magnetic resonance imaging. *J Cereb Blood Flow Metab* 2008;28:1491–501.

28 Strbian D, Durukan A, Pitkonen M, *et al.* The blood-brain barrier is continuously open for several weeks following transient focal cerebral ischemia. *Neuroscience* 2008;153:175–81.

29 Durukan A, Marinkovic I, Strbian D, *et al.* Post-ischemic blood-brain barrier leakage in rats: One-week follow-up by MRI. *Brain Res* 2009;1280:158–65.

30 Abo-Ramadan U, Durukan A, Pitkonen M, *et al.* Post-ischemic leakiness of the blood-brain barrier: A quantitative and systematic assessment by Patlak plots. *Exp Neurol* 2009;219:328–33.

31 Merali Z, Huang K, Mikulis D, *et al.* Evolution of blood-brain-barrier permeability after acute ischemic stroke. *PLoS One* 2017;12:1–11.

32 Liu HS, Chung HW, Chou MC, *et al.* Effects of microvascular permeability changes on contrast-enhanced t1 and pharmacokinetic mr imagings after ischemia. *Stroke* 2013;44:1872–7.

33 Donahue MJ, Achten E, Cogswell PM, *et al.* Consensus statement on current and emerging methods for the diagnosis and evaluation of cerebrovascular disease. *J Cereb Blood Flow Metab* 2018;38:1391–417.

34 Mikulis DJ. Functional cerebrovascular imaging in brain ischemia: Permeability, reactivity, and functional MR imaging. *Neuroimaging Clin N Am* 2005;15:667–80.

35 Yen P, Cobb A, Shankar JJS. Does computed tomography permeability predict

For peer review only

hemorrhagic transformation after ischemic stroke? *World J Radiol* 2016;8:594.

36 Leigh R, Christensen S, Campbell BCV, *et al.* Pretreatment blood-brain barrier disruption and post-endovascular intracranial hemorrhage. *Neurology* 2016;87:263–9.

37 Nguyen GT, Coulthard A, Wong A, *et al.* Measurement of blood-brain barrier permeability in acute ischemic stroke using standard first-pass perfusion CT data. *NeuroImage Clin* 2013;2:658–62.

38 Chen H, Zhu G, Liu N, *et al.* Applications and development of permeability imaging in ischemic stroke (Review). *Exp Ther Med* 2018;16:2203–7.

39 Cuenod CA, Balvay D. Perfusion and vascular permeability: Basic concepts and measurement in DCE-CT and DCE-MRI. *Diagn Interv Imaging* 2013;94:1187–204.

40 Gordon Y, Partovi S, Müller-Eschner M, *et al.* Dynamic contrast-enhanced magnetic resonance imaging: fundamentals and application to the evaluation of the peripheral perfusion. *Cardiovasc Diagn Ther* 2014;4:147–64.

41 Harrigan M, Leonardo J, Gibbons K, *et al.* CT Perfusion Cerebral Blood Flow Imaging in Neurological Critical Care. *Neurocrit Care* 2005;2:352-66.

42 Kassner A, Mandell DM, Mikulis DJ. Measuring Permeability in Acute Ischemic Stroke. *Neuroimaging Clin N Am* 2011;21:315–25.

43 Jaillard A, Cronu C, Durieux A, *et al.* Hemorrhagic Transformation in Acute Ischemic Stroke The MAST-E Study Assia. *Stroke* 1999;30:1326–32.

44 Suh CH, Jung SC, Cho SJ, *et al.* MRI for prediction of hemorrhagic transformation in acute ischemic stroke: a systematic review and meta-analysis. *Acta radiol* 2019;18:284185119887593.

45 Suh CH, Jung SC, Cho SJ, *et al.* Perfusion CT for prediction of hemorrhagic transformation in acute ischemic stroke: a systematic review and meta-analysis. *Eur Radiol* 2019;29:4077–87.

46 Ryu WHA, Avery MB, Dharampal N, *et al.* Utility of perfusion imaging in acute stroke treatment: a systematic review and meta-analysis. *J Neurointerv Surg* 2017;9:1012–6.

47 Liberati A, Altman DG, Tetzlaff J, et al. The PRISMA statement for reporting systematic reviews and meta-analyses of studies that evaluate health care interventions: explanation and elaboration. *PLoS Med* 2009 21;6:e1000100.

48 Higgins J, Green S. Cochrane Handbook for Systematic Reviews of Interventions Version 5.1.0 [updated March 2011]. The Cochrane Collaboration 2011. Available at: www.cochrane-handbook.org.

49 Wells G, Shea B, O'Connell D, *et al.* The Newcastle-Ottawa Scale (NOS) for assessing the quality of nonrandomised studies in meta-analyses. 2008. Available at: http://www.ohri.ca/programs/clinical_epidemiology/oxford.asp. (Accessed: 5th August 2019)

50 Luchini C, Stubbs B, Solmi M, *et al.* Assessing the quality of studies in meta-analyses: Advantages and limitations of the Newcastle Ottawa Scale. *World J Radiol* 2017;5:80–4.

51 Islam MM, Iqbal U, Walther B, *et al.* Benzodiazepine Use and Risk of Dementia in the Elderly Population: A Systematic Review and Meta-Analysis. *Neuroepidemiology* 2016;47:181–91.

For peer review only

52 Higgins J, Thompson S, Deeks J, *et al.* Measuring inconsistency in meta-analyses. *BMJ* 2003;327:557–60.

53 Thanvi BR, Treadwell S, Robinson T. Haemorrhagic transformation in acute ischaemic stroke following thrombolysis therapy: Classification, pathogenesis and risk factors. *Postgrad Med J* 2008;84:361–7.

Legends

Figure 1. Flow-chart diagram presenting the selection process for the studies.

For peer review only

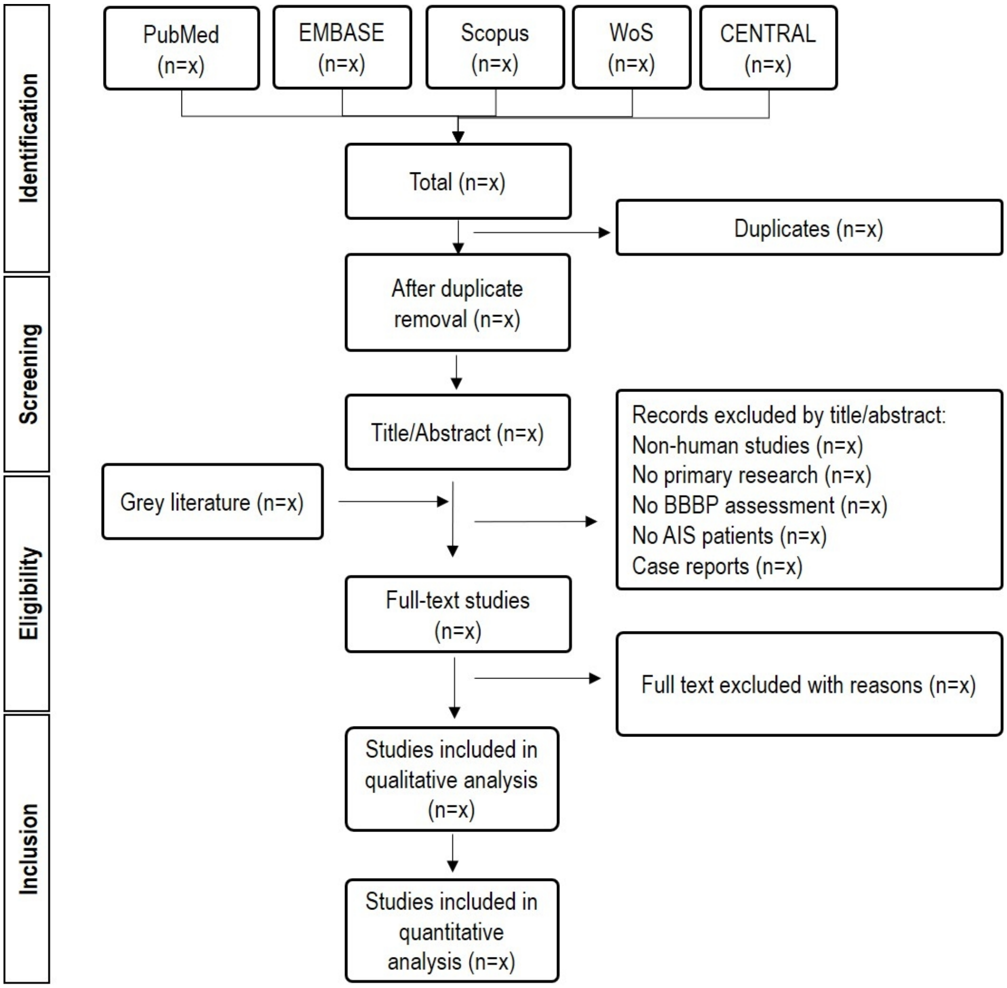

Figure 1. Flow-chart diagram presenting the selection process for the studies.

178x175mm (150 x 150 DPI)

PRISMA-P (Preferred Reporting Items for Systematic review and Meta-Analysis Protocols) 2015 checklist: recommended items to address in a systematic review protocol*

Section and topic	Item No	Checklist item
**ADMINISTRATIVE INFORMATION**		
Title: **pg.1**		
Identification **pg.1**	1a	Identify the report as a protocol of a systematic review
Update **n/a**	1b	If the protocol is for an update of a previous systematic review, identify as such
Registration **pg.2**	2	If registered, provide the name of the registry (such as PROSPERO) and registration number
Authors: **pg.1**		
Contact **pg.1**	3a	Provide name, institutional affiliation, e-mail address of all protocol authors; provide physical mailing address of corresponding author
Contributions **pg.9**	3b	Describe contributions of protocol authors and identify the guarantor of the review
Amendments **pg.9**	4	If the protocol represents an amendment of a previously completed or published protocol, identify as such and list changes; otherwise, state plan for documenting important protocol amendments
Support: **pg.9**		
Sources	5a	Indicate sources of financial or other support for the review
Sponsor	5b	Provide name for the review funder and/or sponsor
Role of sponsor or funder	5c	Describe roles of funder(s), sponsor(s), and/or institution(s), if any, in developing the protocol
**INTRODUCTION**		
Rationale **pg.3**	6	Describe the rationale for the review in the context of what is already known
Objectives **pg.4**	7	Provide an explicit statement of the question(s) the review will address with reference to participants, interventions, comparators, and outcomes (PICO)
**METHODS**		
Eligibility criteria **pg.4**	8	Specify the study characteristics (such as PICO, study design, setting, time frame) and report characteristics (such as years considered, language, publication status) to be used as criteria for eligibility for the review
Information sources **pg.5**	9	Describe all intended information sources (such as electronic databases, contact with study authors, trial registers or other grey literature sources) with planned dates of coverage
Search strategy **pgs.5-6**	10	Present draft of search strategy to be used for at least one electronic database, including planned limits, such that it could be repeated
Study records:		
Data management **pg.6**	11a	Describe the mechanism(s) that will be used to manage records and data throughout the review

For peer review only

Selection process **pg.6**	11b	State the process that will be used for selecting studies (such as two independent reviewers) through each phase of the review (that is, screening, eligibility and inclusion in meta-analysis)
Data collection process **pg.7**	11c	Describe planned method of extracting data from reports (such as piloting forms, done independently, in duplicate), any processes for obtaining and confirming data from investigators
Data items **pg.7**	12	List and define all variables for which data will be sought (such as PICO items, funding sources), any pre-planned data assumptions and simplifications
Outcomes and prioritization **pg.7**	13	List and define all outcomes for which data will be sought, including prioritization of main and additional outcomes, with rationale
Risk of bias in individual studies **pg.8**	14	Describe anticipated methods for assessing risk of bias of individual studies, including whether this will be done at the outcome or study level, or both; state how this information will be used in data synthesis
Data synthesis **pg.8**	15a	Describe criteria under which study data will be quantitatively synthesised
	15b	If data are appropriate for quantitative synthesis, describe planned summary measures, methods of handling data and methods of combining data from studies, including any planned exploration of consistency (such as I^2, Kendall's τ)
	15c	Describe any proposed additional analyses (such as sensitivity or subgroup analyses, meta-regression)
	15d	If quantitative synthesis is not appropriate, describe the type of summary planned
Meta-bias(es) **pg. 9**	16	Specify any planned assessment of meta-bias(es) (such as publication bias across studies, selective reporting within studies)
Confidence in cumulative evidence **pg.9**	17	Describe how the strength of the body of evidence will be assessed (such as GRADE)

*** It is strongly recommended that this checklist be read in conjunction with the PRISMA-P Explanation and Elaboration (cite when available) for important clarification on the items. Amendments to a review protocol should be tracked and dated. The copyright for PRISMA-P (including checklist) is held by the PRISMA-P Group and is distributed under a Creative Commons Attribution Licence 4.0.**

From: Shamseer L, Moher D, Clarke M, Ghersi D, Liberati A, Petticrew M, Shekelle P, Stewart L, PRISMA-P Group. Preferred reporting items for systematic review and meta-analysis protocols (PRISMA-P) 2015: elaboration and explanation. BMJ. 2015 Jan 2;349(jan02 1):g7647.

For peer review only
